# Correction: Characterization of Dietary Patterns in the Danish National Birth Cohort in Relation to Preterm Birth

**DOI:** 10.1371/journal.pone.0099073

**Published:** 2014-05-27

**Authors:** The PLOS ONE Staff

## Notice of Republication

This article was republished on May 6, 2014, to correct the order of Figures 1-3. Please download this article again to view the correct version. The originally published, uncorrected article and the republished, corrected article are provided here for reference.

## Supporting Information

File S1 Originally published, uncorrected article.
(PDF)

File S2 Republished, corrected article.
(PDF)
