# Supplementary material for: Characterization of Dietary Patterns in the Danish National Birth Cohort in Relation to Preterm Birth
Source: PLoS One. 2014 Apr 18;9(4):e93644. doi: 10.1371/journal.pone.0093644 (PMC3991586; doi:10.1371/journal.pone.0093644)
Supplement: Appendix S1 — Dietary pattern analysis, factor modelling and principal component analysis – some details. (DOCX) [file pone.0093644.s001.docx]

**Online Appendix S1**

**Dietary Pattern Analysis**

The purpose of dietary pattern analysis is to use a data-driven approach to derive patterns that can be used to characterize dietary lifestyle. **Figure S1** shows a schematic overview on how the dietary composition can be split into three parts: A) the intake that originates from a certain dietary lifestyle through a combination of dietary patterns; B) the intake that is subject-specific; and C) the uncertainty deriving from the subjective reporting, variation in grocery supply etc. **Figure S1** only shows A (*common part*) and B (*unique* *part*). To make it more explicit, take for example Hannah. In general Hannah eats a lot of different vegetables and different types of seafood. However, tough Hannah eats healthy she also have a craving for diet coke. Hannah hence scores highest for the two dietary patterns *Vegetables* and *Seafood* and low for the *Western* dietary pattern. Hannah’s personal preferences and factors such as seasonality contribute deviations that are unique to Hannah and emphasizes that e.g. Hannah’s intake of diet coke is high compared to other women with a generally similar dietary lifestyle. Decomposing dietary registration surveys into common dietary patterns and person-specific unique parts, results in information that directly can be used for comparison with outcome (as in this work), but also the possibility for adjustment for dietary lifestyle in relations between e.g. a single non-dietary factor (e.g. smoking) and a relevant outcome. The archetype of bi-linear factor models Principal Component Analysis (PCA) is the most common tool used to estimate dietary patterns from food frequency surveys.

**Factor modeling**

Factor modeling is a general tool for analysis of multivariate data, and hence has a broad application range, from micro arrays with translational levels of tenths of thousands of genes to near infrared spectroscopic measures of food samples and dietary surveys. The general idea for all factor models is to truncate the information into a few new meta variables (patterns). Consider a data matrix (**X**) of *n* samples and *p* variables, then the general form is:

**X = TP’ + E** (Equation 1)

Where **T** is an *n* by *k* matrix, **P** is a *p* by *k* matrix and **E** is a residual matrix of the same size as **X**; *k* is the number of patterns extracted. The individual columns of **T** and **P**, together form a pattern/component. Such a pattern/component consists of two parts; one which relates to the variables (**p**) and one which relates to the samples (**t**). In the context of dietary data, **p** shows *which* variables are most responsible for the component and *how* these are correlated, whereas **t** holds the information concerning *how much* each individual person have of this certain pattern. Equation 1 is a bilinear factor model. There exist a variety of methods used for estimation of the parameters (**T** and **P**) depending on which mathematical optimization criterion that is used. PCA is the most general model and PCA solutions obey a set of conditions that makes this model extremely well defined. The two most important ones are: i) given a number of components (*k*), the PCA solution has the *lowest* residual sum of squares and ii) the component matrices in PCA (**T** and **P**) have orthogonal columns. However, PCA models have limitations in terms of interpretation of the results. Therefore different approaches exist for either *post estimation* rotation of parameters (varimax, quatrimax, equimax, direct oblique, and promax) or constrained fitting of the PCA model for example with sparse- or non-negative parameters (sparse PCA, non-negative matrix factorization). In the literature on dietary pattern analysis the predominant method used is PCA with varimax rotation on the variable component matrix (**P**) in order to get patterns that are easier to interpret compared to ordinary PCA.

**Choices and consequences**

PCA is a very well defined method and as such there are not many controllable options. As a matter of fact, the only user-defined choice for unrotated PCA models is whether to initially scale the input variables to unit variance, which for dietary surveys is mandatory. However, in dietary pattern analysis, the PCA model is almost always rotated. The shapes of the estimated patterns of a rotated PCA model rely on the *number* of components (*k*). The number of components is hence a user-defined handle to modify the results. Figure 1 shows the cumulative and individual variance explained per component from an unrotated and a rotated seven component model. The seven components describe 30% of the total data variation. The components of the unrotated model describe from 8.5% to 2.5% of the variance in data, whereas variance explained per component for the rotated model is more equally distributed across the components (from 6.5% to 3%). Where the unrotated PCA solution is *independent* of the total number of component, the rotated model depends on this. For the present example that means that e.g. the most descriptive component of the rotated solution (component 1 – 6.5%) rely on the total number of components (seven) and is hence *not* identical with component 1 from a model with 4 or 9 components. It is therefore important to estimate and further describe the number of components carefully.
