# Supplementary figures and images for: Characterization of Dietary Patterns in the Danish National Birth Cohort in Relation to Preterm Birth
Source: PLoS One. 2014 Apr 18;9(4):e93644. doi: 10.1371/journal.pone.0093644 (PMC3991586; doi:10.1371/journal.pone.0093644)

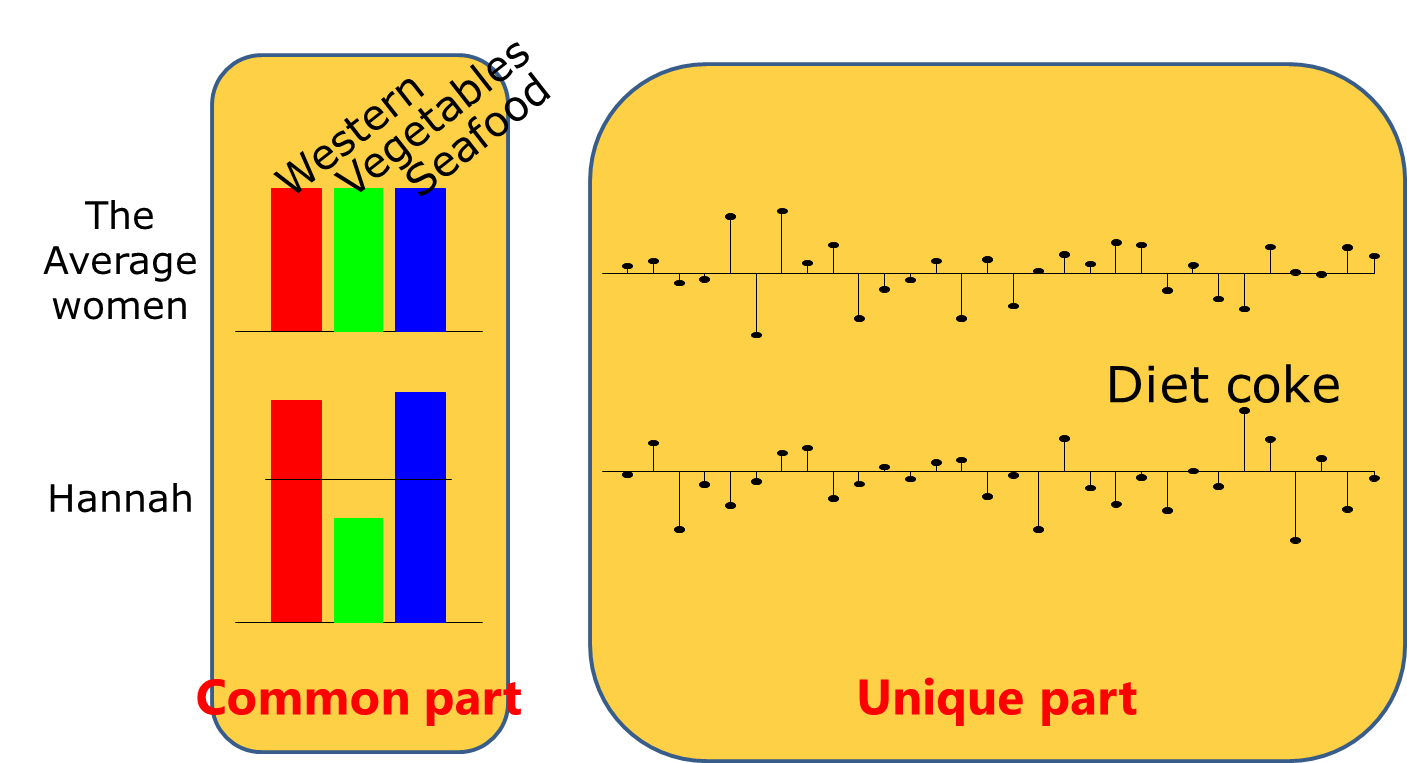

Supplement: Figure S1 — Cartoon of splitting dietary information into a common part reflected by dietary patterns (here Western, Vegetables and Seafood) and a person unique part which cannot be ascribed to general phenomena. (TIF) [file pone.0093644.s002.tif]

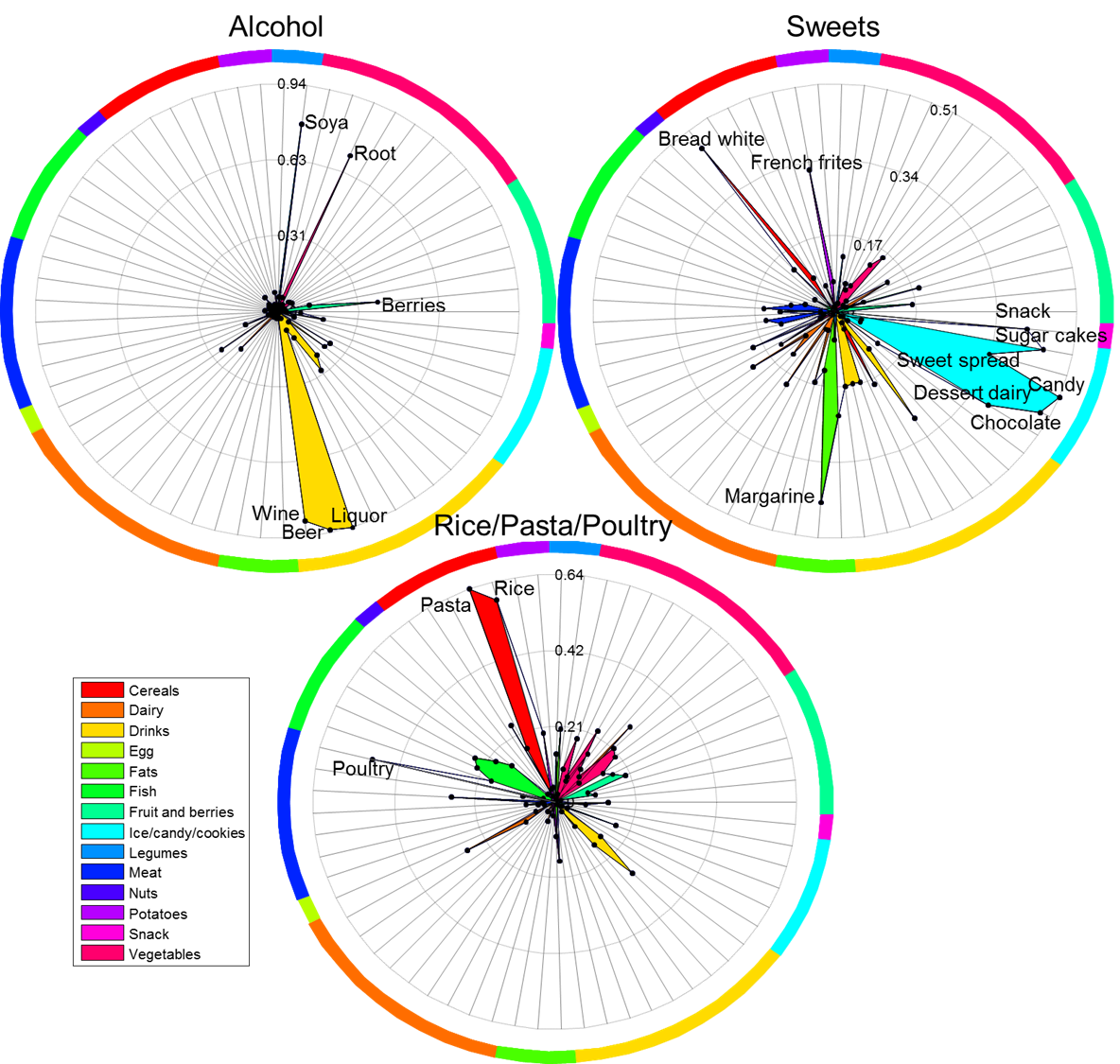

Supplement: Figure S2 — Spider plot(s) of pattern score vs. food item correlations. Upper left – Alcohol, upper right – Sweets, lower– Rice/Pasta/Poultry. The colors refer to food item subgroups. Labels are only shown for food items with a correlation coefficient above 0.3. (TIF) [file pone.0093644.s003.tif]
