# Supplementary material for: Characterization of Dietary Patterns in the Danish National Birth Cohort in Relation to Preterm Birth
Source: PLoS One. 2014 Apr 18;9(4):e93644. doi: 10.1371/journal.pone.0093644 (PMC3991586; doi:10.1371/journal.pone.0093644)
Supplement: File S1 — Tables of food composition of the seven dietary patterns, reflected as average intake in g/day (mean and standard deviation) within pattern score quintiles. (PDF) [file pone.0093644.s004.pdf]

**Vegetables-Prudent**	**1. Quintile**		**2. Quintile**		**3. Quintile**		**4. Quintile**		**5. Quintile**	
**g/day**	**Mean**	**Std**	**Mean**	**Std**	**Mean**	**Std**	**Mean**	**Std**	**Mean**	**Std**
Cabbage	5,4	4,4	9,1	6,6	12,5	8,8	16,7	11,5	27,9	24,2
Onion	7,7	4,7	11,4	6,3	14,2	7,9	17,8	10,1	28,1	20,4
Mushroom	2,3	2,4	3,8	3,6	5,3	4,9	7,6	6,7	14,7	16,6
Corn	1,8	2,4	3,7	3,8	5,5	5,4	8,3	7,6	15,8	16,8
Salad	1,7	1,8	3,1	2,9	4,5	4,4	6,8	6,2	14,0	14,7
Tomato	10,4	7,5	15,7	10,4	20,3	13,5	26,9	18,5	49,7	51,0
Root	7,5	7,7	12,6	11,0	17,4	15,0	23,5	27,4	46,6	67,1
Veg_other	7,2	5,6	12,7	8,5	18,7	11,5	26,8	15,6	54,2	37,2
Asian veg	0,0	0,0	0,0	0,0	0,0	0,0	0,0	0,0	0,0	0,0
Potatoes	105,8	68,4	118,0	72,9	125,9	80,0	133,1	83,1	152,1	112,3
French_frites	9,2	9,7	8,4	8,3	7,8	8,1	7,4	8,1	7,4	10,6
Nuts	1,3	3,2	1,5	3,5	1,8	4,3	2,1	4,9	2,8	6,9
Soya	0,6	16,4	0,5	10,9	0,8	13,1	1,7	28,4	5,8	59,1
Legumes	3,0	3,1	5,5	4,7	8,6	7,0	12,0	9,3	22,1	20,8
Fruit_citrus	35,5	59,7	31,3	50,6	31,3	48,8	31,4	47,7	35,2	50,0
Berries	0,5	1,0	0,6	1,2	0,7	1,4	0,8	1,6	1,0	2,4
Fruit_dried	4,7	12,5	6,2	13,3	7,5	15,1	9,1	16,8	11,9	20,2
Fruit_other	12,3	19,6	22,0	31,4	30,8	43,7	42,4	55,1	61,5	71,3
Banana	17,1	24,0	20,1	24,5	21,9	25,9	23,5	26,0	26,3	26,9
Fruit_nordic	43,0	49,0	54,2	53,7	60,7	55,3	65,2	55,4	75,2	57,5
Poultry	18,6	14,9	22,6	16,8	24,5	17,8	26,8	19,3	30,1	23,0
Pork	24,0	15,4	24,7	15,8	24,3	16,3	23,8	16,5	23,3	19,2
Beef_veal	36,9	21,1	41,3	22,8	41,8	23,2	43,8	24,5	47,8	36,9
Lamb	1,0	3,4	1,5	3,8	1,8	4,4	2,2	4,8	3,0	6,4
Meat_mixed	4,9	4,4	4,8	4,4	4,5	4,2	4,2	4,4	4,2	5,7
Meat_cold	12,7	12,2	11,7	10,9	11,3	10,5	10,9	10,5	10,2	10,9
Offal	0,3	1,6	0,3	1,4	0,3	1,5	0,3	1,3	0,4	2,3
Dessert_dairy	4,2	5,8	4,6	6,0	4,9	6,6	5,1	6,7	5,5	7,9
Chocolate	10,3	12,7	8,9	9,9	8,6	9,9	8,5	9,4	8,5	9,8
Candy	19,2	18,8	18,6	16,9	18,4	17,2	18,5	17,0	18,7	19,2
Sweet_spread	6,0	8,7	6,8	9,0	7,1	9,2	7,7	9,7	8,2	10,1
Sugar_cakes	4,2	4,1	5,2	5,0	5,9	5,6	6,8	6,5	8,6	8,6
Rice	9,5	7,0	10,5	6,9	11,0	7,3	11,7	7,6	13,5	10,1
Pasta	12,3	8,0	13,7	8,1	14,3	8,4	15,2	8,7	16,7	10,5
Breakfast_cereal	26,4	27,1	27,9	26,8	28,8	26,9	30,7	28,0	32,7	30,4
Bread_dark	136,6	80,6	141,0	78,9	148,0	80,5	152,7	83,6	159,0	85,6
Bread_white	94,2	56,9	87,0	51,9	85,0	50,5	83,6	50,3	84,3	53,3
Oil	0,7	1,1	1,0	1,5	1,4	2,0	1,8	2,7	2,9	4,7
Margarine	23,4	21,6	21,2	19,3	20,5	18,7	20,1	18,3	19,9	18,9
Dressing_sauce	4,3	5,4	4,6	5,4	4,7	5,3	5,0	5,8	5,5	7,2
Fish_lean	6,5	7,6	7,6	7,4	8,3	8,2	9,2	8,5	10,7	11,7
Fish_oily	1,9	4,0	2,7	4,8	3,3	5,2	4,1	6,1	5,6	8,1
Shellfish	1,3	2,7	1,4	2,3	1,5	2,4	1,7	2,6	2,1	3,3
Fish_cold	10,1	13,4	10,6	11,6	11,2	12,2	12,0	12,2	14,1	16,9
Fish_smoked	1,4	4,1	1,4	3,0	1,6	3,2	1,8	3,5	2,4	5,0
Wine	9,6	16,0	11,4	16,1	12,0	17,0	13,7	36,3	16,7	65,4
Beer	11,0	39,6	11,6	33,7	11,5	32,3	14,4	118,6	26,6	273,0
Liquor	0,1	0,9	0,1	0,6	0,1	1,3	0,2	4,1	0,6	10,4
Tea	140,1	213,7	144,3	202,3	148,1	203,7	154,7	204,5	166,7	214,3
Coffee	131,0	226,3	141,1	224,7	149,4	227,2	146,4	218,8	152,5	223,5
Softdrink_sugar	191,8	272,0	192,0	268,1	188,3	255,0	199,7	271,3	227,8	339,8
Softdrink_diet	55,9	149,9	58,7	149,4	52,6	138,2	51,5	137,4	55,4	157,0
Water	877,1	506,5	998,7	498,1	1067,5	496,2	1131,9	502,8	1238,4	505,5
Juice	145,2	219,7	167,3	235,4	171,6	229,5	180,1	225,5	216,1	287,8
Milk_lowfat	528,1	402,2	499,2	379,1	495,7	373,5	485,6	376,2	490,6	392,9
Milk_chocolate	53,9	119,1	42,2	91,3	39,0	87,4	36,4	80,7	39,2	98,6
Milk_fermented	2,9	7,1	3,4	7,2	3,8	7,6	4,3	8,4	4,7	9,0
Milk_fullfat	44,1	135,6	44,3	128,1	45,5	130,5	47,6	135,6	51,5	142,4
Yoghurt	40,1	51,7	45,3	51,8	48,8	52,1	52,4	55,3	59,0	61,8
Butter	6,8	8,5	7,4	9,1	7,4	9,5	7,6	9,8	7,9	11,2
Cheese	2,0	6,6	2,3	6,2	2,6	6,1	2,9	6,6	3,4	7,6
Fresh cheese	2,4	5,0	2,9	5,9	3,4	6,5	3,8	7,6	4,9	9,0
Hard cheese	21,9	19,1	23,8	19,4	25,6	19,7	26,4	20,2	28,7	21,1
Snack	4,3	4,6	4,2	4,4	4,1	4,4	3,9	4,4	3,9	5,4
Egg	12,6	9,1	14,4	10,0	15,2	10,2	16,5	11,3	18,7	16,3

**Alcohol**	**1. Quintile**		**2. Quintile**		**3. Quintile**		**4. Quintile**		**5. Quintile**	
**g/day**	**Mean**	**Std**	**Mean**	**Std**	**Mean**	**Std**	**Mean**	**Std**	**Mean**	**Std**
Cabbage	9,8	10,2	12,1	11,5	13,8	12,6	15,7	14,9	20,0	22,0
Onion	13,4	11,8	14,1	11,2	15,1	11,5	16,4	12,7	20,0	17,2
Mushroom	5,4	8,2	5,7	7,7	6,4	8,3	7,0	9,3	9,0	12,9
Corn	7,0	10,8	6,4	8,4	6,7	9,6	6,9	9,0	8,2	12,1
Salad	6,3	10,0	5,4	7,7	5,6	7,8	5,8	7,8	7,0	9,9
Tomato	23,1	39,1	21,5	21,9	22,9	23,6	24,8	23,7	30,6	32,4
Root	10,4	9,0	14,2	11,2	18,0	14,4	22,9	19,1	42,2	72,0
Veg_other	21,3	24,4	20,5	21,1	22,4	22,6	24,3	24,2	31,2	31,9
Asian veg	0,0	0,0	0,0	0,0	0,0	0,0	0,0	0,0	0,0	0,0
Potatoes	121,6	87,0	119,9	77,7	123,8	79,3	127,8	82,6	141,7	100,5
French_frites	7,8	8,3	7,8	7,8	7,9	8,4	8,1	8,7	8,6	11,4
Nuts	1,2	2,4	1,5	2,9	1,7	3,4	2,1	4,4	2,9	8,1
Soya	0,1	0,8	0,1	1,1	0,2	1,8	0,4	2,9	8,5	69,3
Legumes	7,5	10,3	8,6	9,6	9,7	10,9	11,2	12,2	14,3	18,2
Fruit_citrus	21,1	40,7	28,5	47,5	33,4	51,4	38,6	54,8	43,1	58,8
Berries	0,2	0,4	0,4	0,6	0,5	0,8	0,8	1,1	1,6	3,1
Fruit_dried	3,6	7,8	5,4	10,5	7,4	13,1	9,7	17,5	13,3	24,0
Fruit_other	37,3	61,0	32,1	49,4	31,5	46,0	32,4	46,6	35,7	48,8
Banana	20,0	26,2	21,1	25,4	21,7	25,3	22,6	25,1	23,5	26,1
Fruit_nordic	49,4	51,9	56,1	53,6	60,4	54,7	63,8	55,3	68,5	58,9
Poultry	24,2	19,7	24,0	18,4	24,3	18,2	24,7	18,0	25,4	20,3
Pork	23,3	16,4	23,6	15,9	23,8	15,8	24,1	16,1	25,3	19,1
Beef_veal	39,6	23,8	40,8	23,6	41,6	24,2	43,0	25,3	46,6	34,0
Lamb	1,5	4,5	1,6	4,1	1,8	4,3	2,0	4,5	2,6	5,8
Meat_mixed	4,5	4,7	4,4	4,3	4,4	4,3	4,5	4,4	4,7	5,4
Meat_cold	12,0	11,9	11,1	10,9	10,9	10,3	11,2	10,6	11,6	11,6
Offal	0,2	1,0	0,2	1,2	0,3	1,3	0,3	1,6	0,5	2,7
Dessert_dairy	3,5	3,5	4,1	4,1	4,6	4,8	5,3	6,1	6,9	11,2
Chocolate	10,1	12,9	8,4	9,7	8,5	9,4	8,6	9,2	9,2	10,5
Candy	17,7	17,8	17,9	16,7	18,4	16,7	19,2	17,7	20,3	20,2
Sweet_spread	8,4	11,1	6,7	8,9	6,7	8,6	6,8	8,7	7,2	9,3
Sugar_cakes	5,1	5,5	5,4	5,5	5,9	5,9	6,5	6,3	7,8	7,8
Rice	11,2	8,5	11,0	7,6	11,1	7,5	11,2	7,6	11,6	8,5
Pasta	15,1	9,6	14,4	8,7	14,2	8,5	14,3	8,6	14,3	9,2
Breakfast_cereal	22,6	24,4	28,1	26,6	30,1	27,3	32,2	28,5	33,5	31,2
Bread_dark	151,7	86,2	144,7	80,2	143,3	78,8	147,5	80,9	150,2	84,7
Bread_white	100,5	60,7	85,3	50,7	81,2	48,1	82,0	48,4	84,9	52,6
Oil	1,5	3,1	1,4	2,4	1,4	2,5	1,6	2,6	2,0	3,3
Margarine	23,8	23,2	20,0	18,1	19,8	17,8	20,0	17,7	21,5	19,6
Dressing_sauce	5,3	6,9	4,7	5,4	4,5	5,3	4,6	5,3	4,9	6,3
Fish_lean	6,8	7,4	7,8	7,7	8,5	8,3	9,1	8,7	10,2	11,5
Fish_oily	2,9	5,7	3,2	5,3	3,4	5,6	3,7	5,7	4,5	7,0
Shellfish	1,5	2,6	1,5	2,5	1,5	2,5	1,6	2,7	1,9	3,2
Fish_cold	10,0	12,0	10,7	11,8	11,4	12,5	12,1	13,4	13,8	16,7
Fish_smoked	1,3	3,4	1,5	3,4	1,7	3,4	1,8	3,9	2,3	5,0
Wine	3,5	5,4	6,8	7,7	10,0	9,8	14,2	13,3	28,7	75,4
Beer	2,7	8,1	5,2	12,0	8,8	16,6	13,3	22,9	45,1	300,6
Liquor	0,0	0,1	0,0	0,2	0,1	0,3	0,1	0,5	0,9	11,2
Tea	104,8	167,2	133,1	191,4	153,1	204,2	168,0	217,2	194,8	241,1
Coffee	87,0	172,5	119,6	201,3	146,6	221,0	166,0	235,5	201,2	263,0
Softdrink_sugar	117,4	125,6	148,3	167,4	177,4	206,2	223,9	282,6	332,4	454,7
Softdrink_diet	24,2	52,9	36,3	73,0	46,4	95,5	59,1	128,9	108,1	263,3
Water	1011,3	511,9	1038,4	502,2	1060,2	502,1	1080,8	509,2	1123,0	548,3
Juice	99,3	126,0	137,6	158,9	167,2	195,2	206,7	243,1	269,5	370,5
Milk_lowfat	472,5	365,2	504,2	368,9	510,6	374,6	512,0	389,1	499,9	423,9
Milk_chocolate	19,8	34,1	28,3	45,4	35,2	61,8	46,8	87,5	80,5	172,2
Milk_fermented	4,1	8,7	3,6	7,4	3,6	7,4	3,7	7,5	4,1	8,4
Milk_fullfat	21,0	45,4	27,5	68,1	38,5	101,7	55,2	144,7	90,7	222,4
Yoghurt	43,4	52,5	46,8	52,3	48,7	53,2	50,9	54,4	55,8	61,5
Butter	7,5	10,4	6,8	8,4	7,0	8,6	7,4	9,6	8,3	10,9
Cheese	2,0	6,1	2,2	5,9	2,5	6,3	2,9	7,3	3,4	7,5
Fresh cheese	3,4	7,3	3,2	6,5	3,3	6,6	3,6	7,1	4,0	7,4
Hard cheese	25,0	20,8	24,1	19,5	24,4	19,3	25,6	19,8	27,2	20,7
Snack	3,6	3,9	3,9	4,0	4,0	4,3	4,2	4,9	4,7	5,9
Egg	12,9	9,4	14,1	9,6	15,3	10,4	16,3	11,3	18,9	16,2

Western	1. Quintile		2. Quintile		3. Quintile		4. Quintile		5. Quintile	
g/day	Mean	Std	Mean	Std	Mean	Std	Mean	Std	Mean	Std
Cabbage	13,1	13,3	13,3	13,2	13,8	13,8	14,2	13,5	17,1	20,5
Onion	15,9	12,7	15,0	11,9	15,1	11,9	15,2	12,2	18,0	16,9
Mushroom	7,6	10,3	6,6	8,8	6,3	8,3	6,1	8,7	6,9	11,3
Corn	7,8	10,4	6,9	9,3	6,7	9,4	6,6	9,5	7,2	11,6
Salad	8,9	12,3	6,2	8,5	5,3	7,2	4,9	6,8	4,8	6,8
Tomato	30,0	27,7	24,7	29,8	22,4	29,9	21,5	22,6	24,3	33,5
Root	23,3	30,1	20,0	26,8	19,7	27,6	20,4	37,8	24,3	52,4
Veg_other	34,8	32,2	24,7	23,7	21,3	22,1	19,3	21,2	19,5	22,9
Asian veg	0,0	0,0	0,0	0,0	0,0	0,0	0,0	0,0	0,0	0,0
Potatoes	82,8	50,2	100,2	56,2	118,3	65,1	140,7	75,6	192,7	119,3
French_frites	4,3	5,1	6,4	6,2	7,9	7,4	9,3	8,7	12,3	13,3
Nuts	2,7	8,4	1,7	3,1	1,7	3,4	1,6	2,6	1,7	3,5
Soya	2,9	32,4	1,0	14,9	1,0	20,7	1,1	25,2	3,3	50,3
Legumes	9,5	10,7	9,5	11,0	9,8	11,4	10,3	12,5	12,1	17,1
Fruit_citrus	40,0	57,4	33,8	51,7	32,2	51,6	29,8	48,4	28,9	47,4
Berries	0,7	1,8	0,6	1,3	0,7	1,5	0,7	1,4	0,8	1,9
Fruit_dried	14,4	24,0	8,3	15,6	6,5	12,6	5,5	11,4	4,9	10,8
Fruit_other	48,9	63,7	35,1	51,0	31,1	47,0	28,0	44,4	25,9	40,9
Banana	28,8	30,3	23,3	26,4	20,3	24,1	18,6	22,5	17,9	22,7
Fruit_nordic	76,6	61,0	62,5	55,0	56,9	53,8	52,5	51,1	49,8	51,0
Poultry	26,7	20,8	25,2	18,8	23,9	17,5	23,2	17,3	23,6	19,9
Pork	11,4	7,9	17,6	9,4	22,6	11,1	28,3	13,6	40,2	21,4
Beef_veal	29,5	17,9	36,6	19,1	41,3	21,4	46,3	23,3	57,8	37,4
Lamb	2,6	5,5	2,1	4,4	1,8	4,1	1,6	4,0	1,5	5,3
Meat_mixed	1,8	2,0	3,1	2,6	4,1	3,1	5,4	3,8	8,3	6,9
Meat_cold	5,1	5,2	8,0	6,9	10,4	8,4	13,3	10,1	19,9	15,4
Offal	0,1	0,6	0,2	0,9	0,2	1,0	0,3	1,4	0,8	3,1
Dessert_dairy	5,0	8,0	4,8	5,8	4,8	5,7	4,7	6,0	5,2	7,5
Chocolate	8,8	11,2	8,7	10,0	8,8	10,3	8,9	10,0	9,5	10,7
Candy	18,1	17,6	18,0	16,9	18,2	16,8	18,7	17,2	20,4	20,6
Sweet_spread	5,7	7,8	6,2	8,2	6,8	8,9	7,6	9,7	9,5	11,4
Sugar_cakes	6,3	6,6	5,8	5,9	5,8	5,8	6,1	6,1	6,7	7,1
Rice	11,6	7,9	11,1	7,4	11,0	7,4	10,9	7,4	11,6	9,4
Pasta	14,9	8,7	14,5	8,6	14,2	8,4	14,1	8,7	14,5	10,1
Breakfast_cereal	36,4	29,7	30,7	27,7	27,6	26,4	25,9	26,1	25,9	28,3
Bread_dark	125,9	74,6	138,2	78,1	144,9	80,4	153,9	80,8	174,3	88,7
Bread_white	58,4	35,4	71,9	39,8	83,4	44,5	97,3	50,0	123,0	64,3
Oil	2,4	3,9	1,6	2,7	1,4	2,3	1,2	2,0	1,2	2,7
Margarine	12,1	10,1	15,8	12,9	19,2	15,2	23,8	18,4	34,3	27,6
Dressing_sauce	2,5	2,7	3,4	3,3	4,1	3,9	5,3	4,9	8,8	9,5
Fish_lean	7,5	7,1	7,8	7,2	8,1	7,4	8,6	7,9	10,3	13,2
Fish_oily	5,2	6,9	3,6	5,5	3,2	5,1	2,8	4,9	2,9	6,6
Shellfish	1,4	2,4	1,4	2,3	1,5	2,3	1,6	2,6	2,1	3,6
Fish_cold	7,7	7,8	9,2	8,7	10,5	10,0	12,3	12,0	18,3	21,4
Fish_smoked	1,3	2,3	1,4	2,4	1,5	2,8	1,7	3,5	2,7	6,6
Wine	12,3	18,0	11,8	16,4	11,9	27,6	12,5	36,3	14,7	61,2
Beer	8,8	31,0	9,7	24,0	12,0	71,3	15,6	134,9	28,9	259,7
Liquor	0,1	0,4	0,1	0,5	0,1	3,0	0,2	5,0	0,6	9,6
Tea	182,5	227,6	158,0	206,7	142,4	196,2	139,9	201,9	131,1	202,0
Coffee	89,9	147,9	114,9	181,0	133,3	204,7	162,8	237,6	219,5	297,4
Softdrink_sugar	168,8	241,6	182,2	255,2	195,1	264,5	207,1	287,3	246,2	348,7
Softdrink_diet	36,4	96,9	45,9	117,6	55,3	140,8	61,1	155,8	75,5	198,0
Water	1179,2	494,2	1073,5	505,4	1033,6	504,0	1005,1	514,7	1022,4	543,5
Juice	169,3	211,9	168,9	217,9	169,8	221,9	174,8	242,1	197,6	303,1
Milk_lowfat	441,3	345,7	487,7	356,1	506,8	374,6	522,7	393,0	540,7	441,5
Milk_chocolate	30,7	71,8	35,1	78,4	39,8	85,6	46,1	98,8	59,1	133,0
Milk_fermented	4,6	9,6	3,8	8,1	3,5	7,1	3,5	7,1	3,7	7,5
Milk_fullfat	19,9	55,3	26,6	75,4	37,0	104,6	49,3	129,5	100,3	223,6
Yoghurt	56,1	58,7	49,4	52,9	46,9	52,7	45,6	52,3	47,5	57,6
Butter	4,1	5,8	5,5	7,2	6,8	8,2	8,3	9,5	12,3	13,6
Cheese	3,2	7,8	2,7	6,7	2,4	5,6	2,4	6,2	2,4	6,9
Fresh cheese	3,8	7,7	3,3	6,6	3,1	6,3	3,3	6,3	3,9	7,8
Hard cheese	22,8	18,8	24,1	19,1	24,7	19,5	26,0	20,2	28,8	21,9
Snack	2,5	2,8	3,4	3,3	3,9	3,8	4,6	4,6	6,0	6,8
Egg	11,2	7,9	13,0	8,6	14,7	9,4	16,5	10,4	22,0	17,3

Nordic	1. Quintile		2. Quintile		3. Quintile		4. Quintile		5. Quintile	
g/day	Mean	Std	Mean	Std	Mean	Std	Mean	Std	Mean	Std
Cabbage	10,5	11,6	12,3	11,9	13,9	14,2	15,9	15,5	18,9	20,0
Onion	13,3	11,6	14,3	11,6	15,4	12,1	16,9	13,9	19,2	16,0
Mushroom	6,2	10,2	6,4	8,4	6,5	8,5	7,1	9,8	7,4	10,6
Corn	6,5	10,2	6,8	9,0	7,1	9,9	7,3	10,4	7,5	10,8
Salad	5,4	7,9	5,7	8,3	5,9	8,4	6,3	8,8	6,8	10,0
Tomato	20,9	27,0	22,4	31,2	23,7	26,8	26,3	29,3	29,6	30,1
Root	14,5	29,1	17,3	26,2	20,5	33,3	24,3	38,1	30,9	48,2
Veg_other	17,4	19,5	20,8	21,7	22,9	23,5	26,6	26,7	31,8	31,5
Asian veg	0,0	0,0	0,0	0,0	0,0	0,0	0,0	0,0	0,0	0,0
Potatoes	115,2	80,4	118,2	78,6	123,5	78,0	130,7	85,4	147,2	102,2
French_frites	12,1	12,6	8,6	8,2	7,5	7,6	6,5	7,3	5,5	6,7
Nuts	1,4	2,7	1,5	2,7	1,7	3,2	2,0	4,3	2,8	8,3
Soya	1,5	29,0	1,1	21,1	1,7	27,9	2,5	36,6	2,6	38,3
Legumes	8,1	11,5	9,2	10,7	10,0	12,3	11,1	12,8	13,0	15,7
Fruit_citrus	14,8	27,9	24,4	42,3	32,8	51,0	40,5	56,2	52,2	64,4
Berries	0,4	0,9	0,5	1,0	0,6	1,2	0,8	1,5	1,2	2,7
Fruit_dried	2,7	6,1	4,3	8,1	6,1	10,4	8,9	14,2	17,5	27,0
Fruit_other	26,4	47,7	31,9	50,4	35,4	52,2	37,0	51,8	38,4	50,4
Banana	9,9	12,7	15,3	17,5	20,7	22,5	26,8	27,0	36,1	34,3
Fruit_nordic	27,4	30,7	44,0	42,7	58,3	50,2	74,0	56,5	94,4	64,1
Poultry	23,8	19,6	24,1	18,5	24,6	18,4	25,0	19,0	25,0	19,2
Pork	22,8	15,6	23,2	15,5	23,9	16,2	24,4	17,0	25,8	18,9
Beef_veal	43,2	26,8	41,4	24,9	41,2	24,9	42,1	26,8	43,6	29,1
Lamb	1,5	4,5	1,7	4,5	1,8	4,6	2,1	4,6	2,4	5,3
Meat_mixed	5,0	5,0	4,6	4,4	4,4	4,3	4,4	4,6	4,4	4,8
Meat_cold	7,2	6,6	9,3	8,1	10,9	9,6	12,8	11,2	16,7	15,3
Offal	0,2	1,6	0,2	1,2	0,3	1,5	0,3	1,7	0,4	2,1
Dessert_dairy	5,2	7,4	4,8	7,1	4,7	5,7	4,8	6,5	4,9	6,5
Chocolate	8,3	9,9	8,3	9,9	8,6	9,7	9,2	10,5	10,2	11,9
Candy	21,3	21,3	19,1	17,3	18,3	16,6	17,6	16,2	17,2	17,2
Sweet_spread	4,0	5,1	5,2	6,6	6,5	8,1	8,1	9,7	12,0	13,1
Sugar_cakes	4,3	3,9	5,1	4,7	5,8	5,6	6,7	6,4	8,9	8,9
Rice	10,3	7,8	10,8	7,6	11,2	7,6	11,7	8,1	12,2	8,5
Pasta	13,8	8,8	14,2	8,7	14,4	8,6	14,8	9,0	15,0	9,3
Breakfast_cereal	21,1	22,2	26,1	25,0	29,0	26,4	32,0	28,7	38,3	33,2
Bread_dark	76,3	51,9	118,1	57,6	144,3	62,1	174,1	67,3	224,4	82,8
Bread_white	68,5	45,3	78,8	46,3	85,5	48,7	93,0	51,6	108,1	61,7
Oil	1,4	3,0	1,5	2,6	1,5	2,6	1,6	2,9	1,8	2,9
Margarine	15,5	12,7	18,3	15,1	20,1	16,9	22,6	19,3	28,7	27,3
Dressing_sauce	4,7	5,6	4,7	5,5	4,7	5,4	4,9	6,0	5,1	6,8
Fish_lean	6,6	7,6	7,7	7,6	8,4	8,1	9,3	9,3	10,3	11,1
Fish_oily	3,2	6,1	3,4	5,8	3,5	5,7	3,7	6,0	3,8	6,0
Shellfish	1,8	3,3	1,6	2,5	1,5	2,4	1,6	2,5	1,6	2,7
Fish_cold	7,9	9,2	9,6	9,8	11,0	11,0	13,0	13,2	16,5	19,7
Fish_smoked	1,2	2,8	1,5	2,9	1,7	3,5	1,9	4,1	2,3	5,4
Wine	11,3	38,1	12,0	25,3	12,7	35,9	13,7	40,0	13,5	37,9
Beer	13,8	133,9	12,8	100,4	14,4	124,5	16,2	143,4	17,8	168,7
Liquor	0,2	4,7	0,2	3,4	0,2	4,8	0,3	5,8	0,3	6,1
Tea	89,3	151,5	120,6	174,5	147,1	195,6	174,9	221,5	222,0	255,3
Coffee	104,0	198,2	134,0	214,3	146,3	223,6	158,9	230,9	177,2	244,7
Softdrink_sugar	291,1	391,8	202,3	273,2	183,2	250,9	165,9	227,4	157,1	215,8
Softdrink_diet	80,3	207,8	58,1	145,5	52,2	129,8	45,1	118,4	38,5	105,7
Water	833,8	504,9	991,2	498,6	1078,1	494,5	1152,6	486,1	1258,0	495,1
Juice	168,3	256,3	168,0	227,5	173,3	232,3	178,0	233,2	192,8	257,9
Milk_lowfat	428,3	373,7	477,7	368,4	505,2	374,8	522,9	381,5	565,3	412,4
Milk_chocolate	64,6	137,2	43,3	95,3	36,3	79,0	34,1	76,5	32,3	76,7
Milk_fermented	2,9	5,8	3,2	6,6	3,6	7,3	4,3	8,8	5,1	10,2
Milk_fullfat	47,8	138,1	42,9	126,9	42,0	120,8	46,4	133,8	53,9	150,9
Yoghurt	38,5	46,4	44,1	50,4	47,9	51,9	53,9	57,2	61,1	64,7
Butter	7,1	8,1	6,9	8,1	7,1	9,1	7,5	10,2	8,3	12,1
Cheese	1,1	2,9	1,7	3,9	2,2	4,8	3,0	6,3	5,0	11,2
Fresh cheese	1,8	3,6	2,5	4,5	3,1	5,6	4,0	7,1	6,0	10,9
Hard cheese	15,1	12,6	19,8	16,1	24,5	18,8	29,1	20,6	37,8	22,6
Snack	5,6	6,4	4,3	4,7	3,8	3,9	3,5	3,8	3,1	3,5
Egg	13,6	10,3	14,5	11,0	15,2	10,9	16,1	12,2	18,1	13,9

Seafood	1. Quintile		2. Quintile		3. Quintile		4. Quintile		5. Quintile	
g/day	Mean	Std	Mean	Std	Mean	Std	Mean	Std	Mean	Std
Cabbage	10,5	12,6	12,1	12,0	13,7	13,3	15,5	14,2	19,7	20,7
Onion	10,6	8,9	13,0	10,6	15,1	11,1	17,5	12,7	23,0	17,9
Mushroom	3,9	6,9	5,1	7,3	6,4	8,6	7,7	9,5	10,3	12,9
Corn	5,4	10,0	6,0	8,7	6,9	9,8	7,5	9,6	9,2	11,6
Salad	3,4	5,1	4,6	6,4	5,7	7,7	6,8	9,0	9,7	12,2
Tomato	14,2	13,3	18,6	16,6	22,5	19,7	27,7	25,2	39,9	48,5
Root	16,2	26,9	17,7	24,6	19,9	30,9	23,4	41,0	30,4	49,9
Veg_other	13,2	16,3	17,5	18,8	21,8	21,7	27,6	25,1	39,4	33,1
Asian veg	0,0	0,0	0,0	0,0	0,0	0,0	0,0	0,0	0,0	0,0
Potatoes	116,9	78,7	120,1	79,6	124,7	80,7	128,6	82,8	144,4	103,7
French_frites	9,2	9,1	8,6	9,1	7,9	8,2	7,4	8,1	7,1	10,2
Nuts	0,9	1,8	1,2	2,2	1,6	2,6	2,1	3,7	3,6	8,9
Soya	0,3	11,1	0,5	12,2	1,3	25,1	2,1	34,8	5,2	52,5
Legumes	9,0	14,2	8,9	11,1	9,5	11,3	10,6	11,5	13,2	15,0
Fruit_citrus	25,2	47,5	30,8	51,6	32,9	51,9	36,1	52,1	39,6	53,4
Berries	0,3	0,7	0,5	0,9	0,6	1,2	0,8	1,6	1,2	2,7
Fruit_dried	3,1	8,0	4,9	10,0	7,2	14,3	10,0	17,9	14,2	22,7
Fruit_other	20,4	37,3	27,8	46,3	33,0	49,4	38,8	53,3	49,1	59,8
Banana	19,7	27,3	20,2	24,9	21,4	24,8	22,9	25,2	24,6	25,7
Fruit_nordic	53,2	57,3	56,3	55,9	58,5	54,2	63,2	54,1	67,0	54,0
Poultry	17,1	14,6	21,8	16,6	25,0	18,0	27,6	19,0	31,1	22,5
Pork	26,0	16,6	24,9	15,8	24,2	15,8	22,9	15,6	22,0	19,2
Beef_veal	35,7	20,3	39,7	21,5	42,6	23,2	43,8	24,4	49,8	37,5
Lamb	0,2	1,0	0,6	1,8	1,2	2,7	2,2	3,8	5,4	8,2
Meat_mixed	4,8	4,4	4,7	4,4	4,6	4,3	4,3	4,1	4,2	5,7
Meat_cold	13,4	13,3	11,2	10,5	11,0	10,1	10,7	10,0	10,4	10,9
Offal	0,1	0,5	0,1	0,7	0,2	1,1	0,4	1,5	0,7	3,1
Dessert_dairy	4,2	5,5	4,5	6,6	4,8	6,9	5,1	6,5	5,8	7,6
Chocolate	8,5	10,2	8,7	10,4	8,8	10,0	9,2	10,6	9,5	10,9
Candy	18,9	19,2	18,8	17,2	18,7	17,1	18,5	17,3	18,5	18,6
Sweet_spread	6,7	9,8	6,6	9,0	7,0	9,1	7,4	9,3	8,0	9,6
Sugar_cakes	4,2	4,4	5,2	5,3	6,0	6,0	6,8	6,5	8,5	8,0
Rice	9,9	7,5	10,6	7,3	11,1	7,5	11,6	7,7	13,0	9,3
Pasta	13,2	8,8	13,9	8,6	14,4	8,6	14,9	8,6	15,8	9,7
Breakfast_cereal	19,5	22,3	26,2	25,2	29,3	27,1	32,9	28,3	38,6	32,1
Bread_dark	138,0	84,6	138,5	79,9	145,8	79,5	153,0	80,5	162,0	84,0
Bread_white	96,1	59,3	86,2	52,6	83,9	49,1	82,6	48,9	85,2	52,1
Oil	0,5	0,7	0,8	1,1	1,1	1,5	1,7	2,2	3,7	5,0
Margarine	22,7	22,0	20,6	18,9	20,5	18,2	20,4	18,3	21,0	19,4
Dressing_sauce	3,4	4,0	4,3	4,7	4,9	5,3	5,1	5,7	6,3	8,3
Fish_lean	3,5	4,2	6,1	5,5	8,1	6,5	10,3	7,6	14,3	13,5
Fish_oily	0,6	1,7	1,5	2,8	2,7	3,8	4,5	5,2	8,4	9,3
Shellfish	0,6	0,9	1,0	1,2	1,4	1,7	1,9	2,3	3,3	4,7
Fish_cold	5,9	6,6	8,8	8,3	11,1	10,3	13,3	12,3	18,9	21,0
Fish_smoked	0,4	1,0	0,9	1,6	1,3	2,1	2,0	3,0	4,0	7,0
Wine	7,5	14,3	9,9	21,3	12,5	33,4	14,6	40,9	18,7	53,9
Beer	9,1	63,1	10,8	54,9	13,6	122,8	16,8	158,0	24,7	213,0
Liquor	0,1	1,8	0,1	2,2	0,2	4,4	0,3	6,0	0,4	8,0
Tea	92,9	160,5	130,9	196,4	151,2	204,2	174,8	219,6	203,9	234,3
Coffee	167,2	267,5	140,4	224,7	139,7	216,7	137,4	207,6	135,7	196,5
Softdrink_sugar	190,1	282,9	189,9	267,1	196,7	275,0	198,9	270,6	223,9	316,3
Softdrink_diet	96,9	228,6	55,3	129,8	46,7	114,8	40,4	110,2	34,9	102,7
Water	1006,6	543,3	1024,2	516,5	1049,9	505,5	1082,6	500,1	1150,3	502,9
Juice	144,9	234,4	162,6	230,5	172,9	235,0	187,5	233,9	212,5	268,4
Milk_lowfat	549,3	419,5	515,7	388,2	498,7	372,2	483,0	368,0	452,6	368,9
Milk_chocolate	53,8	122,3	42,9	95,3	39,7	85,0	37,2	82,9	36,9	90,9
Milk_fermented	1,4	2,4	2,1	3,7	3,2	5,9	4,6	8,4	7,8	12,8
Milk_fullfat	29,2	94,2	39,4	119,7	47,0	135,7	51,1	143,3	66,3	166,2
Yoghurt	23,8	32,2	37,8	43,6	48,3	50,4	59,3	56,5	76,4	69,7
Butter	5,6	6,0	6,6	7,3	7,0	8,1	7,9	10,2	9,9	14,1
Cheese	0,7	2,1	1,3	3,2	2,1	4,5	3,1	6,5	5,8	11,4
Fresh cheese	1,7	3,9	2,5	4,7	3,2	5,7	4,1	7,2	6,0	10,6
Hard cheese	22,0	20,0	23,7	19,6	24,9	19,5	26,7	19,8	29,0	20,7
Snack	4,9	5,5	4,2	4,4	4,0	4,5	3,7	4,0	3,6	4,8
Egg	10,1	6,5	13,1	7,9	15,1	9,4	17,3	11,1	21,9	17,4

Sweets	1. Quintile		2. Quintile		3. Quintile		4. Quintile		5. Quintile	
g/day	Mean	Std	Mean	Std	Mean	Std	Mean	Std	Mean	Std
Cabbage	19,0	20,8	14,3	13,8	13,3	12,9	12,4	12,1	12,5	13,7
Onion	16,5	14,9	15,1	12,1	15,2	12,3	15,4	12,0	16,9	14,8
Mushroom	6,8	9,8	6,4	8,8	6,4	8,7	6,6	9,2	7,4	11,0
Corn	5,5	8,4	5,9	7,8	6,5	9,1	7,4	9,6	9,8	13,8
Salad	4,5	6,6	5,2	7,2	5,9	8,2	6,5	9,1	7,9	11,3
Tomato	23,0	26,8	23,0	24,0	23,9	25,2	24,5	23,7	28,6	41,3
Root	25,9	37,9	20,6	25,4	19,7	23,2	18,9	23,1	22,6	58,3
Veg_other	22,4	24,1	22,3	23,4	23,2	24,1	24,3	25,2	27,3	29,4
Asian veg	0,0	0,0	0,0	0,0	0,0	0,0	0,0	0,0	0,0	0,0
Potatoes	126,8	86,3	120,8	77,9	122,4	77,8	126,2	83,7	138,6	101,8
French_frites	4,6	5,1	6,4	6,2	7,7	7,1	9,1	8,6	12,4	13,5
Nuts	1,4	2,7	1,5	2,9	1,8	3,8	1,9	3,9	2,8	8,1
Soya	1,8	28,1	1,1	15,1	1,1	17,6	0,8	11,6	4,6	58,3
Legumes	12,6	16,4	10,0	11,5	9,7	11,9	9,2	10,9	9,8	12,3
Fruit_citrus	46,0	64,0	34,6	52,5	30,7	48,2	27,5	44,2	25,9	43,9
Berries	0,5	1,0	0,6	1,2	0,6	1,2	0,7	1,4	1,0	2,7
Fruit_dried	9,4	18,5	7,9	15,6	7,6	15,3	7,3	14,6	7,3	15,5
Fruit_other	21,9	31,7	27,3	39,1	31,8	46,2	38,4	54,8	49,7	68,7
Banana	20,9	25,0	21,1	24,8	21,7	25,7	22,2	25,9	23,0	26,7
Fruit_nordic	63,5	59,3	60,3	56,1	59,4	55,2	57,9	52,6	57,1	52,9
Poultry	22,6	17,5	23,8	17,9	24,4	18,4	25,1	19,1	26,6	21,4
Pork	22,4	15,9	23,0	15,4	23,6	15,6	24,4	16,4	26,7	19,6
Beef_veal	37,6	24,2	39,7	22,8	41,8	23,5	44,1	25,8	48,5	33,7
Lamb	1,8	4,5	1,8	4,1	1,9	4,3	2,0	4,7	2,2	5,8
Meat_mixed	3,7	3,9	4,1	3,9	4,4	4,2	4,8	4,5	5,6	6,0
Meat_cold	9,9	10,2	10,4	10,1	11,0	10,5	11,9	11,1	13,5	12,9
Offal	0,5	2,6	0,3	1,3	0,2	1,2	0,2	1,3	0,2	1,6
Dessert_dairy	2,7	2,7	3,6	3,2	4,4	4,0	5,3	5,1	8,4	11,9
Chocolate	3,8	3,8	5,8	5,2	7,7	6,9	10,5	8,9	16,9	16,6
Candy	9,3	7,8	13,5	10,1	17,4	12,6	21,6	15,0	31,7	27,5
Sweet_spread	3,5	4,6	5,0	6,0	6,3	7,5	8,3	9,6	12,6	13,6
Sugar_cakes	3,3	2,8	4,4	3,5	5,5	4,3	6,9	5,6	10,7	9,9
Rice	11,2	8,2	11,2	7,6	11,1	7,4	11,2	7,7	11,4	8,8
Pasta	13,6	8,6	14,1	8,6	14,4	8,4	14,8	8,9	15,3	10,0
Breakfast_cereal	39,1	31,0	31,2	27,4	28,1	26,4	25,5	25,6	22,6	26,1
Bread_dark	137,3	77,3	141,7	77,7	147,4	80,7	152,0	82,3	158,8	90,8
Bread_white	57,4	29,7	70,4	36,6	82,4	42,8	97,2	50,2	126,6	67,1
Oil	1,1	1,9	1,3	2,1	1,5	2,4	1,7	2,9	2,2	4,1
Margarine	11,9	9,3	15,3	11,8	19,3	15,0	23,8	18,4	34,8	28,1
Dressing_sauce	3,2	4,1	4,0	4,5	4,6	5,2	5,4	5,9	6,9	8,2
Fish_lean	9,4	10,2	8,2	7,7	8,2	8,1	8,1	8,2	8,3	10,1
Fish_oily	3,9	6,4	3,5	5,6	3,4	5,6	3,4	5,5	3,5	6,5
Shellfish	1,5	2,8	1,5	2,4	1,6	2,7	1,7	2,6	1,8	3,0
Fish_cold	14,0	16,6	11,2	11,8	10,9	11,9	10,8	12,1	11,1	14,0
Fish_smoked	2,3	5,7	1,6	3,1	1,6	3,1	1,5	3,0	1,6	3,8
Wine	9,5	14,8	11,4	18,2	12,0	16,3	12,8	18,7	17,5	72,3
Beer	9,5	36,6	10,6	28,0	11,7	32,5	12,4	34,5	30,7	296,4
Liquor	0,1	0,5	0,1	0,8	0,1	0,6	0,1	0,9	0,8	11,2
Tea	141,2	195,5	147,7	201,8	148,9	203,6	155,2	212,0	160,9	225,1
Coffee	143,5	224,1	141,5	217,8	142,1	219,4	141,0	220,9	152,3	238,2
Softdrink_sugar	114,6	153,8	152,7	202,7	185,8	241,0	226,0	289,6	320,4	411,6
Softdrink_diet	39,0	95,6	46,7	117,3	54,1	143,3	57,7	148,2	76,6	203,1
Water	1064,0	511,4	1055,1	505,4	1058,0	507,7	1062,3	518,3	1074,3	538,4
Juice	151,8	220,8	160,5	215,7	171,5	228,2	182,0	238,3	214,5	293,6
Milk_lowfat	605,1	416,3	511,4	371,1	481,8	361,2	462,3	367,1	438,7	386,0
Milk_chocolate	22,9	48,2	30,5	60,3	38,7	82,2	48,0	100,9	70,5	149,4
Milk_fermented	2,8	6,9	3,5	7,5	3,9	7,8	4,2	8,3	4,7	8,8
Milk_fullfat	31,0	102,0	38,0	115,7	43,4	127,9	49,4	136,7	71,2	175,4
Yoghurt	50,0	59,0	48,0	52,5	48,7	52,7	49,0	54,1	49,8	56,6
Butter	5,0	6,2	6,3	7,6	7,2	8,9	8,2	10,1	10,3	13,1
Cheese	2,2	5,4	2,4	6,0	2,6	6,4	2,8	7,1	3,0	8,1
Fresh cheese	3,2	6,7	3,3	6,6	3,3	6,5	3,6	6,9	4,0	8,1
Hard cheese	20,7	17,7	23,0	18,5	25,1	19,4	27,1	20,5	30,5	22,4
Snack	2,2	2,3	3,1	2,8	3,8	3,2	4,5	4,0	6,8	7,6
Egg	13,1	9,9	14,0	9,9	15,1	10,4	16,1	11,2	19,1	15,7

Rice-Pasta-Poultry	1. Quintile		2. Quintile		3. Quintile		4. Quintile		5. Quintile	
g/day	Mean	Std	Mean	Std	Mean	Std	Mean	Std	Mean	Std
Cabbage	12,5	13,1	13,0	13,4	13,6	12,9	14,8	14,7	17,6	20,1
Onion	13,7	11,8	14,3	11,4	14,9	11,3	16,5	13,2	19,7	17,0
Mushroom	4,7	6,3	5,5	7,0	6,2	7,4	7,3	9,3	9,9	14,5
Corn	6,3	9,9	6,5	9,5	6,7	9,0	7,2	9,4	8,5	12,1
Salad	4,8	8,0	5,5	8,3	5,9	8,2	6,5	8,8	7,4	9,9
Tomato	18,3	19,9	20,8	20,0	23,6	26,3	26,4	25,2	33,8	44,3
Root	20,3	51,4	19,0	30,8	19,9	26,7	22,2	29,7	26,2	36,9
Veg_other	18,5	22,2	21,1	22,6	23,1	22,6	26,0	25,5	30,9	31,2
Asian veg	0,0	0,0	0,0	0,0	0,0	0,0	0,0	0,0	0,0	0,0
Potatoes	163,1	101,9	131,4	79,8	118,7	71,4	111,7	70,8	109,9	91,3
French_frites	6,4	6,8	7,1	7,1	7,8	7,7	8,7	8,7	10,3	12,9
Nuts	1,8	5,8	1,7	3,8	1,8	4,6	2,0	4,4	2,1	4,8
Soya	3,2	48,5	1,4	24,6	1,4	22,1	1,4	23,8	2,0	29,4
Legumes	8,8	10,5	9,2	11,8	9,5	10,6	10,8	12,7	13,0	17,0
Fruit_citrus	22,2	39,2	27,9	46,4	33,0	50,6	38,1	55,6	43,4	60,7
Berries	1,0	2,6	0,7	1,3	0,6	1,2	0,6	1,2	0,6	1,2
Fruit_dried	5,5	12,4	6,6	13,4	7,8	15,1	9,2	17,6	10,5	19,8
Fruit_other	27,0	47,6	31,1	48,8	34,6	51,1	36,3	50,6	40,0	54,3
Banana	14,5	18,8	18,0	21,3	21,5	24,1	25,4	27,4	29,5	31,8
Fruit_nordic	44,7	47,7	53,0	51,4	60,2	54,1	67,6	57,7	72,7	60,2
Poultry	14,3	10,6	18,6	12,1	22,8	13,9	27,8	17,3	39,1	26,0
Pork	25,9	17,2	24,1	15,7	23,5	15,2	22,8	15,3	23,9	19,6
Beef_veal	35,4	21,3	38,3	21,5	41,2	22,0	44,8	24,5	51,9	37,0
Lamb	1,9	4,9	2,0	4,5	1,9	4,2	1,9	4,3	1,9	5,5
Meat_mixed	4,8	4,3	4,5	4,1	4,4	4,1	4,3	4,4	4,7	5,9
Meat_cold	11,6	11,1	11,0	10,5	11,1	10,5	11,0	10,7	12,1	12,4
Offal	0,5	2,2	0,3	1,5	0,3	1,6	0,2	1,2	0,3	1,8
Dessert_dairy	4,8	7,3	4,8	6,3	4,8	6,0	4,9	5,7	5,1	7,8
Chocolate	8,4	10,1	8,7	9,6	8,9	10,1	9,2	10,6	9,5	11,7
Candy	14,7	14,3	16,9	14,9	18,1	15,4	20,2	18,4	23,5	23,5
Sweet_spread	8,0	10,8	7,2	9,4	6,8	8,8	6,9	8,9	6,8	8,8
Sugar_cakes	7,3	8,2	6,2	6,3	5,9	5,7	5,7	5,4	5,6	5,6
Rice	6,5	4,6	8,7	4,6	10,2	5,0	12,1	6,5	18,7	11,0
Pasta	8,4	5,1	11,0	5,2	13,1	6,0	16,3	7,7	23,5	10,7
Breakfast_cereal	23,2	25,6	26,5	25,7	29,2	26,8	32,0	28,2	35,6	31,4
Bread_dark	151,0	87,5	146,7	81,1	145,4	80,0	146,0	80,7	148,2	81,6
Bread_white	99,3	60,3	87,0	51,3	83,2	49,0	81,4	48,6	83,0	51,7
Oil	1,6	3,4	1,5	2,6	1,5	2,5	1,5	2,4	1,6	3,1
Margarine	29,1	25,7	22,3	19,4	19,3	16,7	17,7	15,6	16,8	15,2
Dressing_sauce	4,6	5,4	4,5	5,0	4,5	5,1	4,7	5,5	5,6	7,8
Fish_lean	7,2	7,3	7,9	7,5	8,4	7,6	8,9	8,3	9,9	12,6
Fish_oily	2,3	4,2	2,9	4,7	3,5	5,3	4,0	5,8	5,0	8,4
Shellfish	1,0	1,5	1,2	1,8	1,5	2,0	1,8	2,6	2,5	4,3
Fish_cold	9,2	9,9	9,9	10,0	10,9	10,9	12,0	12,4	15,9	20,2
Fish_smoked	1,2	2,5	1,4	2,6	1,6	3,1	1,7	3,2	2,6	6,4
Wine	15,2	65,0	13,2	31,1	12,3	23,7	11,8	19,4	10,8	17,0
Beer	28,2	261,8	14,6	107,6	11,9	77,4	10,7	62,4	9,5	48,0
Liquor	0,7	10,0	0,2	4,1	0,1	2,5	0,1	1,7	0,1	0,6
Tea	153,3	224,7	156,3	211,7	150,9	203,5	148,4	197,0	144,9	201,6
Coffee	248,9	300,2	154,8	219,3	126,0	197,3	103,0	175,2	87,6	165,1
Softdrink_sugar	202,0	313,5	194,7	271,4	189,8	255,3	193,2	256,6	219,9	312,3
Softdrink_diet	31,0	95,5	38,8	98,7	47,6	115,1	61,4	147,0	95,3	226,2
Water	851,8	492,8	961,5	481,1	1064,6	485,9	1153,8	493,6	1282,0	518,0
Juice	135,8	209,9	150,8	200,6	173,4	227,1	191,7	235,4	228,7	310,1
Milk_lowfat	346,7	332,9	456,9	341,8	507,3	356,3	556,2	380,4	632,2	443,3
Milk_chocolate	33,0	82,4	36,6	82,0	39,4	83,7	43,9	94,1	57,6	129,7
Milk_fermented	5,3	10,6	4,1	8,3	3,5	7,0	3,2	6,6	2,9	6,1
Milk_fullfat	124,9	250,7	36,3	94,3	27,2	70,6	22,9	57,4	21,7	52,0
Yoghurt	48,4	55,4	48,4	52,9	48,2	52,7	50,0	55,4	50,6	58,5
Butter	11,1	14,8	7,4	8,6	6,6	7,5	6,0	6,7	5,9	7,5
Cheese	3,5	9,4	2,8	6,5	2,5	5,8	2,3	5,4	2,0	5,3
Fresh cheese	3,2	6,6	3,3	6,9	3,3	6,4	3,6	6,9	4,1	8,0
Hard cheese	27,0	21,4	25,3	19,9	24,9	19,6	24,3	19,3	24,9	19,9
Snack	3,7	4,2	3,9	4,0	4,0	4,2	4,2	4,4	4,7	6,1
Egg	16,1	11,9	15,1	10,4	15,0	10,2	15,1	10,4	16,2	15,4
