# Supplementary material for: Correction: Characterization of Dietary Patterns in the Danish National Birth Cohort in Relation to Preterm Birth
Source: PLoS One. 2014 May 27;9(5):e99073. doi: 10.1371/journal.pone.0099073 (PMC4035333; doi:10.1371/journal.pone.0099073)
Supplement: File S2 — Republished, corrected article. (PDF) [file pone.0099073.s002.pdf]

OPEN ACCESS Freely available online

PLOS ONE

Characterization of Dietary Patterns in the Danish National Birth Cohort in Relation to Preterm Birth

Morten Arendt Rasmussen[1]*, Ekaterina Maslova[2], Thorhallur Ingi Halldorsson[2,3], Sjurdur Frodi Olsen[2,4]

1 Faculty of Science, University of Copenhagen, Frederiksberg, Denmark, **2** Centre for Fetal Programming, Division of Epidemiology, Statens Serum Institut, Copenhagen, Denmark, **3** The Unit for Nutrition Research, Faculty of Food Science and Nutrition, School of Health Sciences, University of Iceland, Reykjavik, Iceland, **4** Department of Nutrition, Harvard School of Public Health, Boston, Massachusetts, United States of America

Abstract

Background: Dietary patterns better reflect eating habits as opposed to single dietary components. However, the use of dietary pattern analysis in nutritional epidemiology has been hampered by the complexity of interpreting and presenting multidimensional dietary data.

Methods: This study extracts and visualizes dietary patterns from self-reported dietary data collected in mid-pregnancy (25th week of gestation) from nearly 60,000 mother-child pairs part of a prospective, longitudinal cohort (Danish National Birth Cohort) and further examines their associations with spontaneous and induced preterm birth (gestational age<259 days (<37 weeks)).

Results: A total of seven dietary patterns were extracted by principal component analysis, characterized and visualized by color-coded spider plots, and referred to as: Vegetables/Prudent, Alcohol, Western, Nordic, Seafood, Candy and Rice/Pasta/Poultry. A consistent dose-response association with preterm birth was only observed for Western diet with an odds ratio of 1.30 (95% CI: 1.13, 1.49) comparing the highest to the lowest quintile. This association was primarily driven by induced preterm deliveries (odds ratio = 1.66, 95% CI: 1.30, 2.11, comparing the highest to the lowest quintile) while the corresponding odds ratio for spontaneous preterm deliveries was more modest (odds ratio = 1.18, 95% CI: 0.99, 1.39). All based on adjusted analyses.

Conclusions: In conclusion, this study presented a simple and novel framework for visualizing correlation structures between overall consumption of foods group and their relation to nutrient intake and maternal characteristics. Our results suggest that Western-type diet, high in meat and fats and low in fruits and vegetables, is associated with increased odds of induced preterm birth.

Citation: Rasmussen MA, Maslova E, Halldorsson TI, Olsen SF (2014) Characterization of Dietary Patterns in the Danish National Birth Cohort in Relation to Preterm Birth. PLoS ONE 9(4): e93644. doi:10.1371/journal.pone.0093644

Editor: Martin Gerbert Frasch, Université de Montréal, Canada

Received August 30, 2013; **Accepted** March 6, 2014; **Published** April 18, 2014

Copyright: © 2014 Rasmussen et al. This is an open-access article distributed under the terms of the Creative Commons Attribution License, which permits unrestricted use, distribution, and reproduction in any medium, provided the original author and source are credited.

Funding: This study was supported by the Danish Council for Strategic Research (09-067124); the Danish Council for Independent Research | Medical Sciences, Danish Agency for Science, Technology and Innovation (09-063410); the Lundbeck foundation (R13-A907); and the European Union (EU) Integrated Research Project EARNEST (FOOD-CT-2005-007036). The EU project EARNEST (http://www.metabolic-programming.org) receives financial support from the Commission of the European Communities under the FP 6 priority 5: food quality and safety. The Danish National Birth Cohort has been financed by the March of Dimes Birth Defects Foundation, the Danish Heart Association, the Danish Medical Research Council, and the Sygekassernes Helsefond, Danish National Research Foundation, Danish Pharmaceutical Association, Ministry of Health, National Board of Health, Statens Serum Institut. The supporting bodies for this project had no role in the design, analysis or writing of this article.

Competing Interests: The authors have declared that no competing interests exist.

* E-mail: mortenr@food.ku.dk

Introduction

Traditional analysis of the relation between diet and an outcome is often conducted based on a single or a few food items or nutrients. However, dietary surveys from observational cohorts consist of correlated data exhibiting an underlying structure of distinct (or multiple) dietary patterns. Intuitively these dietary patterns characterize a dietary lifestyle where e.g. high intake of salad greens is related to high intake of tomatoes and other vegetables. Furthermore, outcomes of interest related to dietary habits, such as specific diseases or anthropometrical measures, are complex and multivariate in nature, and the interplay between diet and environment relevant for understanding the cause of a certain pathology.

Principal component analysis (PCA) is a well-established and commonly used method for empirically deriving dietary patterns by reducing the dimensionality of the original data. PCA constructs dietary patterns based on correlations among individual food items and is not driven by the relation to any specific health outcome. Dietary pattern analysis is not a novel technique, but has proven successful in the understanding of nutritional data and its relation to various maternal and child outcomes [1]. Diet scores have been constructed using principal components analysis, cluster, and latent variable analysis in a variety of populations, including British [2], Spanish [3], Norwegian [4], and Japanese [5] populations. These scores have been evaluated against pregnancy and offspring outcomes, including preeclampsia [6], gestational diabetes [7], birth weight [8] and fetal growth [9,10],

spina bifida [11], child wheeze/asthma [12–14], child bone mass [15], and, pediatric tumors [16]. For further details on dietary pattern analysis and factor modeling see online **Appendix S1**.

Few studies have examined maternal dietary patterns in relation to length of gestation. Two large Scandinavian cohort studies [17,18], including the Danish Nation Birth Cohort (DNBC), assessed adherence to a Mediterranean diet during pregnancy in relation to preterm birth where only the Danish study found a reduced risk [18]. A Norwegian clinical trial that randomized 290 women to either a 'health conscious' diet or continuing their usual diet, found that the intervention reduced risk of preterm birth [19].

The aim of the present work was to estimate and characterize dietary patterns in the DNBC in novel ways using PCA. A secondary aim was to characterize the individual patterns in relation to demographic information and relate them to preterm birth (gestational age (GA) <259 days (<37 weeks)). In this context we present a simple framework based on intuitive visualizations of correlation structures between food consumptions, nutrient intake, and maternal characteristics.

Methods

The Danish National Birth Cohort

The DNBC is a prospective, longitudinal cohort study of prenatal and early life exposures, and diseases in the offspring. Women were enrolled between January 1996 and October 2002 and all women living in Denmark, who could speak Danish and were planning to carry to term, were eligible for recruitment. About 60% of all eligible women received an invitation from their general practitioner and of those 60% chose to participate. More than 100,000 pregnancies were enrolled. The women participated in two telephone interviews during pregnancy at 12 and 30 weeks of gestation and 6 and 18 months postpartum. A food frequency questionnaire (FFQ) was administered in mid-pregnancy. The mother-child dyads have been followed-up through national registries linkages using their unique identity (CPR) number.

A total of 91,827 pregnant women registered into the cohort and women were allowed to enter the study repeatedly during the study period. However, we restricted our analyses to first pregnancy enrollment to avoid using dependent observations. Of these 91,827 women 70,188 filled out the FFQ. Women who reported unrealistic low energy intake estimates (arbitrarily set to <5000 kJ/day, approximately 1%) were excluded. A final study sample of 69,305 pregnant women was used in the estimation of the dietary patterns. In the comparative analysis with preterm birth we further restricted our analyses to singleton pregnancies and children with a gestational age between 200 and 330 days. The lower boundary excludes 62 (1.0 ‰) pregnancies where many are assumed to reflect miscarriages. According to clinical guidelines women are given medicine to induce labor when reaching week 42. Here we restricted our analysis to women who delivered prior to gestational week 47 assuming errors in recording the GA variable – no women were excluded due to this criterion. Due to this inclusion criterion and missing information on gestational age we ended up with a total of 59,949 women in our final data or 65% of all subjects recruited. The pregnant women provided written informed consent on behalf of their children. The Regional Scientific Ethics Committee for the municipalities of Copenhagen and Frederiksberg approved all study protocols, and all procedures were in accordance with the Declaration of Helsinki.

Dietary assessment

All enrolled women were sent a validated semi-quantitative FFQ around the 25th week of gestation. The FFQ asked about intake of about 360 different food items in the past four weeks. These records where translated into 65 food groups. Details of dietary calculations have been described elsewhere [20]. Briefly, nutrient intakes were calculated by multiplying daily frequencies with standardized portions and using Danish food tables. We energy-adjusted all nutrient intakes using the residual method [21]. We report here intake of foods in grams/day and nutrient intakes using the most appropriate unit for that nutrient (http://www.foodcomp.dk/v7/fvdb_nutrlist.asp - March, 2013). For this analysis we utilized all dietary data on 65 foods to construct the dietary pattern components.

Assessment of maternal characteristics

We extracted information on maternal and lifestyle characteristics from the two pregnancy interviews. The following self-reported factors were evaluated as potential confounders, and entered as categorical variables: urbanity (Capital and suburbs, population >100.000, 10.000< population <99.999, population <9.999), maternal age (<20, 20–39, ≥40; 0% missing), pre-pregnancy BMI (≤18.5, 18.6–24.9, 25–29.9, 30–35, >35 in kg/m^2; 6% missing), parity (0, 1, 2+; 4% missing), civil status (single, coupled/married; 4% missing), parental socioeconomic status (high, medium, skilled, student, unskilled, unemployed; 7% missing), physical activity level (inactive, light, moderate, high; 5% missing), smoking during pregnancy (non-smoker, occasional smoker, <15 cigarettes/day, > = 15 cigarettes/day; 1% missing), and alcohol consumption (yes/no; 1% missing), energy intake (in quartiles; 0% missing), and intake of dietary supplements (yes/no; 1% missing) and preferences for organic foods (categories; 0% missing) during pregnancy.

Presentation of results

Dietary patterns were depicted as correlations between individual food items and dietary factor score. These are shown as spider plots where the 65 food items are partitioned and colored according to 14 predefined food groups. Comparison of dietary patterns and maternal characteristics were done by cross-tabulation between quintilized dietary factor scores and demographic categories. These results are depicted as circles with diameter proportional to the fraction, and the degree of deviation from independency as colors on a blue-red color scale. Plotting were conducted using in-house routines for Matlab. The spider plot routine is available at www.models.life.ku.dk/dietarypattern

Preterm assessment

For assessing length of gestation, date of birth was extracted from the Danish Civil Registration System. Gestational age in days was then assessed from the last menstrual period based on information from the recruitment form (in week 6) and telephone interview (in week 12). If this estimate was uncertain due to irregular or abnormally long (>32 days) or short (<24 days) menstrual cycles, gestational age was based on expected date of delivery provided by the women in the second telephone interview (week 30), which is most often based on ultrasound scanning. If neither of these two sources were available, we used the gestational age as reported by the midwife to the Medical Birth Registry. For our gestational age estimates 43%, 56% and 1% were based on information on last menstrual period, information from the second telephone interview, and the Medical Birth Registry respectively. Preterm birth was defined as GA<259 days (<37 weeks), whereas

early preterm birth, late preterm birth and very late preterm birth were defined as GA<224 days (<32 weeks), GA<266 days (<38 weeks), and GA<273 days (<39 weeks) respectively. Preterm birth cases were stratified on type birth; spontaneous (65%) and induced (33%).

Statistical analysis

PCA was used to derive a set of principal components from the 65 food items (details on the method used can be found in **Appendix S1**). In order not to favor food items with numerical high registrations, the items were scaled to unit variance. The extracted principal components were rotated using the varimax criterion [22]. The number of factors (seven) were decided based on i) scree plot of eigenvalues, ii) eigenvalues above 1 and iii) interpretability of the (rotated) factors. For each extracted dietary factor, quintile scores were created in order to allow for non-linear associations. The association with preterm birth was assessed using multivariate logistic regression by comparisons between quintiles of the pattern scores using the lowest quintile as reference and the pattern scores as a continuous predictor. The first test, tests whether all five quintiles could be considered equal, which is a test where the pattern score consumes four degrees of freedom, and is hence reported as P_{df4}, whereas the latter test is referred to as trend test and reported as P_{trend}. Results are presented as odds ratios with 95% confidence interval. The most significant associations were further investigated for interaction (effect modification) with confounders using both logistic regression with interaction terms and stratified on the confounder levels. All analyses were conducted in Matlab (R2011a v. 7.12.0.635). Dietary patterns were estimated using the PLS toolbox (v. 6.5.1 - Eigenvector Research inc.) and logistic regression were performed using the glmfit function from the Statistics Toolbox (v. 7.5).

Results

Baseline characteristics of the cohort

Majority of women eligible for this analysis were between 21 and 39 years old (98%), nulliparous (53%), of medium level of proficiency or unskilled (51%), and lived in settlements of >10,000 inhabitants (60%). About 61% of women reported no physical activity during pregnancy and 25% reported either occasional or daily smoking. Use of supplements was common with 94% of women reporting use and 54% consumed alcohol (average 2.8 g/day) during pregnancy (**Figure 1** – top panel).

Characterization of dietary patterns

In total, seven principal components were estimated, explaining 30.6% of the total variation in the data. The components were varimax rotated in order to get simpler and more intuitive patterns without sacrificing the components ability to describe the data. **Figure 2** shows the explained variance (per component and cumulated) for both the unrotated and the rotated solution. The rotated components are named based on the food items with high factor correlations. This resulted in the following components (%variation explained): Alcohol (6.4%), Vegetable/Prudent (5.4%), Western (4.9%), Seafood (4.6%), Nordic (3.2%), Sweets (3.2%), and Rice/Pasta/Poultry (3.0%) dietary patterns.

Figure 3 shows spiderplots of the correlations between each individual food item and the pattern factor scores for four of the seven patterns. In order to facilitate interpretation the 65 food items are partitioned into 14 subgroups and colored according to these. In the component Vegetable/Prudent the food items *Cabbage*, *Onion*, *Mushroom*, *Corn*, *Salad*, *Tomato* and *Vegetables other* (from the vegetable subgroup) and *Legumes* obtain factor correlations above 0.5 and naturally found the basis for the name of this component. Likewise for the Western component the most abundant food items were: *Potatoes*, *French fries*, *Bread white*, *Pork*, *Beef Veal*, *Meat mixed*, *Meat cold* and *Dressing sauce*, whereas the Nordic component is reflected by *Bread dark* and to some extend *Fruit nordic* and *Hard cheese*. The Seafood component mainly associated with the food item partition *Fish*. The remaining three patterns are shown in **Figure S2**. In the online **Supplementary materials** the average intake (±deviation) of the individual food items and nutrients for each dietary factor quintile is shown (**Table S1–S7 in File S1**).

Dietary patterns vs. maternal characteristics

We examined the associations between the maternal characteristics (urbanity, energy intake, physical activity, parity, maternal prepregn ancy BMI, maternal age, parental socioeconomic status, maternal smoking, maternal alcohol intake, and intake of dietary supplements) and quintilized dietary pattern scores. **Figure 1** shows the cross-tabulation frequencies, where the diameter of the dots is proportional to the relative frequency of the dietary quintile within each demographic strata. The colors indicate deviation from independence, such that blue and red indicate over- and under representation respectively. Lighter cross-tables represent no apparent association between maternal characteristics and the corresponding dietary pattern. We see that the derived dietary patterns are to some extent mirrored in the maternal characteristics. For example there is a strong age signature across almost all dietary patterns. Older women tended to consume more of the Seafood dietary pattern and less of the Rice/Past/Poultry diet, while younger women had higher intake of the Western-type diet and low consumption of the Nordic dietary pattern. Energy intake was directly related to the Western, Nordic, and Sweets dietary patterns. Furthermore, eating a Western-type diet was associated with being unskilled worker or unemployed, multiparous, smoking daily during pregnancy, and living in a town with a population <10,000; while it was inversely related to moderate and high physical activity. Consumption at the higher end of the Seafood dietary pattern was directly related to high level proficiency, high level of physical activity, and living in the capital or nearby suburbs, while it was inversely associated with a prepregnancy BMI≥30 kg/m^2.

Dietary patterns vs. preterm birth

Of the 59,949 children, 2,682 (4.5%) were preterm, of these 1,756, 895 and 31 were registered as spontaneous-, induced births or missing on type of delivery, respectively. The latter 31 cases were excluded from the analysis. **Figure 4** shows the association between individual dietary patterns and preterm birth. We found that high intake of Western-type diet was associated with increased odds of induced preterm birth (odds ratio = 1.70, 95% CI: 1.37, 2.11: P_{df4}<0.001) comparing the highest and lowest quintile. This association was relatively unaffected when adjusting for confounders. We found no relation for Western-type diet with spontaneous preterm birth in the unadjusted analysis (odds ratio = 1.00, 95% CI: 0.86, 1.17: P_{df4}>0.2). However, adjusting for relevant confounders showed a modest association (P_{df4} = 0.02) with an odds ratio of 1.18, (95% CI: 0.99, 1.39) comparing the highest and lowest quintile. The covariates that were most significant as determinants of risk of preterm birth included: parity (inverse), mother's height (inverse), maternal pre-pregnancy BMI (normal range BMI was protective), maternal age (direct), and smoking (inverse). For further details please consult **Table 1**.

The Seafood dietary pattern showed a tendency towards a moderate protective association in our fully adjusted models (odds

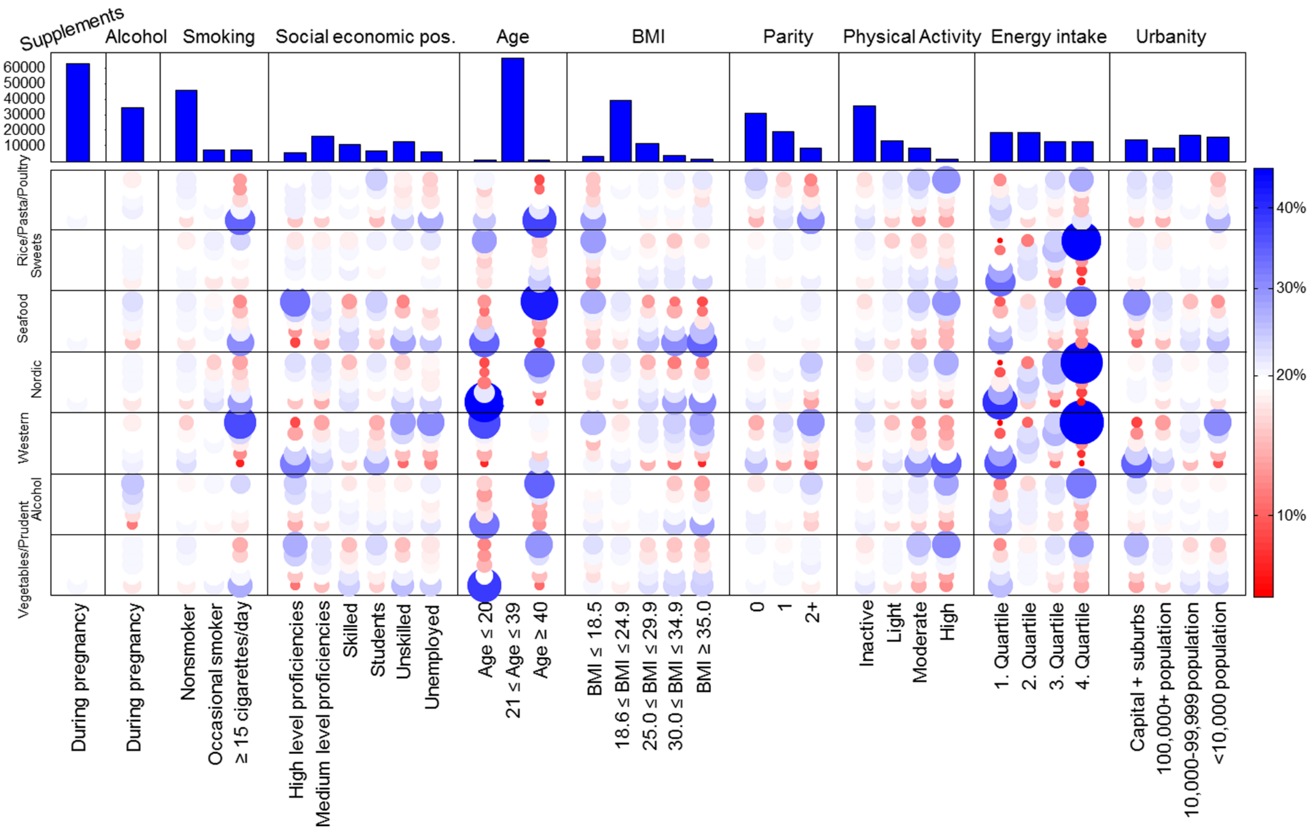

Figure 1. Maternal characteristics vs. dietary patterns. Quintilized dietary pattern scores and single maternal characteristics are compared using cross-tabulation. These cross-tables are depicted as colored circles where the color refers to the distribution of the socio-demographic factor within the dietary pattern. Top panel shows the distribution of the individual maternal covariates.
doi:10.1371/journal.pone.0093644.g001

ratio = 0.62, 95% CI: 0.50, 0.79 comparing quintile four to quintile one). The association was, however, not linear and the adjusted OR comparing quintile five to one was (odds ratio = 0.90, 95% CI: 0.72, 1.11). No association was observed between the seafood dietary pattern and spontaneous preterm birth (see **Figure 4**). The foods and nutrients most correlated with Western and Seafood diet, respectively, were analyzed univariately against preterm birth. None of these were found to be associated with preterm birth independent of Western-type- and Seafood diet to the same extend as the underlying dietary pattern, and a number of both food and nutrients showed an opposite protective association when adjusted for the corresponding dietary pattern (results not shown). This suggests that the association between individual dietary pattern (Western- or Seafood diet) and preterm birth may be driven by clustering of foods where each item has a very modest contribution to the overall odds of preterm birth, but the overall pattern results in significantly increased odds. A separate analysis where the definition of preterm birth was changed to early preterm (GA<32 weeks), late preterm (GA<38 weeks), and very late preterm (GA<39 weeks), revealed the same overall trend between Western-type diet and both induced and spontaneous preterm birth regardless of the preterm definition (results not show). In our study population 26% (233/895) and 6.3% (56/895) of pregnant women with induced preterm birth experienced preeclampsia (PE) and confirmed or suspected cases of gestational diabetes mellitus (GDM), respectively, compared to 4.3% (75/1756) and 2.9% (50/1756) among those with spontaneous preterm birth. When excluding all 1666 PE and 1002 GDM pregnancies from the analysis, we found no substantial changes in the results as the unadjusted odds ratio for induced preterm birth comparing quintile five with one for Western diet was: Odds ratio = 1.63, 95% CI: 1.26, 2.10 ($P_{\mathrm{trend}}<0.001$) (results for the other dietary patterns are likewise unaffected).

The interaction between the Western dietary pattern and maternal age, prepregnancy BMI, parity, and maternal smoking were analyzed by both linear models with interaction terms on the entire dataset and by stratification where a sequence of local models corresponding to each level of the covariate was compared. The results indicated that the dietary effect is additive why there was no interaction- or effect modification by the covariate levels (results not shown).

Discussion

In this large, prospective cohort of close to 60,000 women followed during pregnancy we extracted and described seven dietary patterns using novel visual tools. We further related these dietary components to preterm birth and found that high intake of a Western-type diet increased odds of induced preterm birth, while a Seafood diet was modestly protective of induced preterm birth. Although same directionality was observed for spontaneous preterm birth for Western diet, those associations were much more modest.

In our study the association between Western-type diet and induced preterm birth was relatively consistent with respect to the definition of preterm birth (early or late or very late preterm). Unlike the association observed for Seafood the association with

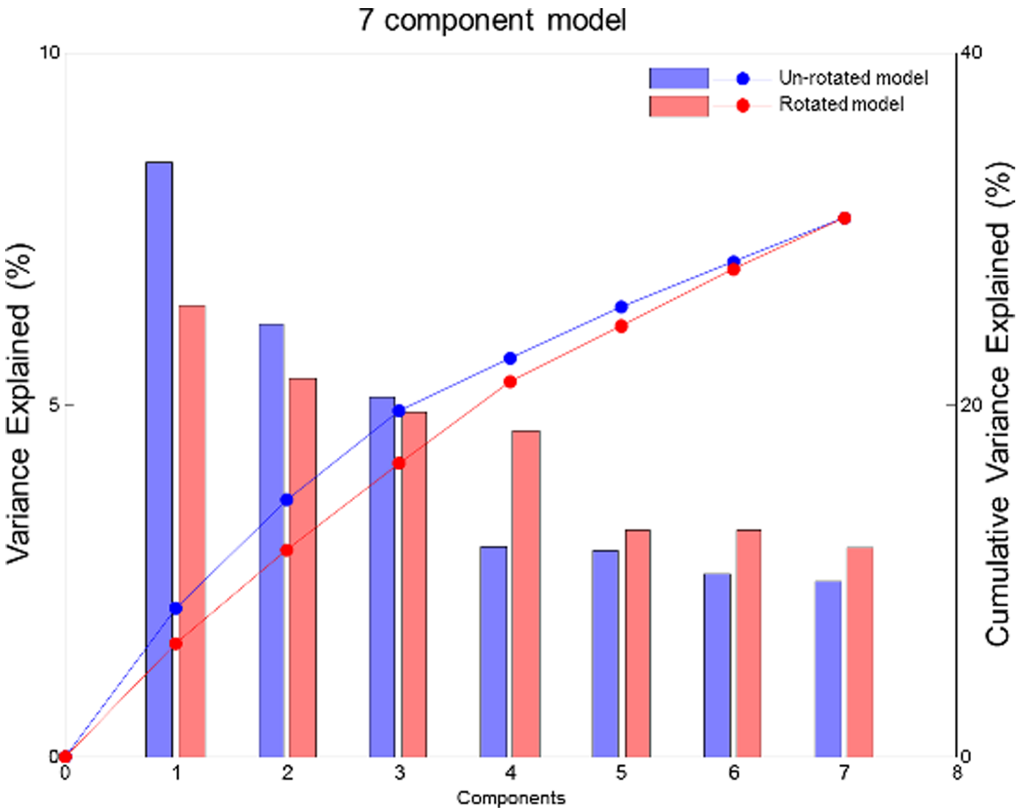

Figure 2. Scree plot of cumulative- (-0-) and per component (bars) variance explained for a unrotated- (blue) and a rotated (red) seven component model.
doi:10.1371/journal.pone.0093644.g002

Western-type diet was also monotonic and strengthened after confounder adjustment. The fact that stronger association was observed for induced rather than spontaneous birth suggests that there may be one or more diagnosed complications that are driving the association [23] including preeclampsia and gestational diabetes that accounted for 26% and 6% of induced preterm deliveries in our sample, respectively. Although the association between Western-type diet and induced preterm birth was stable after excluding these cases, we observed in our stability analyses a significant association between Western-type diet and preeclampsia (data not shown). According to Danish registries the incidence of pregnancy-induced hypertension without proteinuria, also a known cause of induced preterm birth [23], is at least two fold higher than the incidence of preeclampsia [24]. It is also likely that some preeclampsia cases were not covered by our registry based information. Therefore, it cannot be excluded that pregnancy-induced hypertensive disorders may account for the association we observed for Western-type diet. To confirm this would require more rigorous analyses including date of diagnoses of preeclampsia cases and suspected hypertensive cases in relation to the date of dietary assessment, which is beyond the scope of this paper.

In fact, the primary aim of our analyses was to extract and compress information on dietary patterns extracted from a large set of variables (n = 65) and present this information graphically in a format that would be simple and easy to communicate. In this way we can more easily distribute new knowledge, put it into context, and subsequently create a foundation for thinking. However, there is a tradeoff between, on the one hand, how easily results are communicated and, on the other hand, how fair the results are in mirroring the true underlying complexity of the analyzed system. Visualizations can serve as a messenger for presenting fairly complex results in an intuitive manner, preserving some of the underlying complexity without sacrificing the communicability of the results. Although analysis of epidemiological data via pattern recognition tools may be etiologically sound, one bottleneck in the process from project to publication is the presentation of results. Here we used spider plots for presenting dietary patterns, where the 65 food items were arranged and colored according to food group partition. Furthermore, the pattern scores across all seven components were examined across relevant maternal characteristics by cross-tabulation using circles with varying diameter and a red/blue color scale for highlighting deviation from independence – all in a single figure. In our opinion both techniques lead to results that are easier to interpret compared to e.g. multiple cross-tabulations in presenting the same results.

One reason why dietary pattern analyses have not gained wider popularity is that comparison of dietary patterns between different studies requires the ability to judge from tables whether correlation structure in data A is comparable to the correlation structure in data B and to estimate if the food groups used in both studies are actually comparable. Visualization as presented in this study may facilitate better comparison across studies; we have therefore made the software used freely available. However, this by itself does not solve other problems in cross-study comparisons, such as what type of information that should be presented in the results.

A number of studies have examined the role of individual foods and nutrients with respect to their possible effect on risk of preterm birth, but few factors seems to have been confirmed in randomized controlled trials (RCT). One such factor may be the long-chain

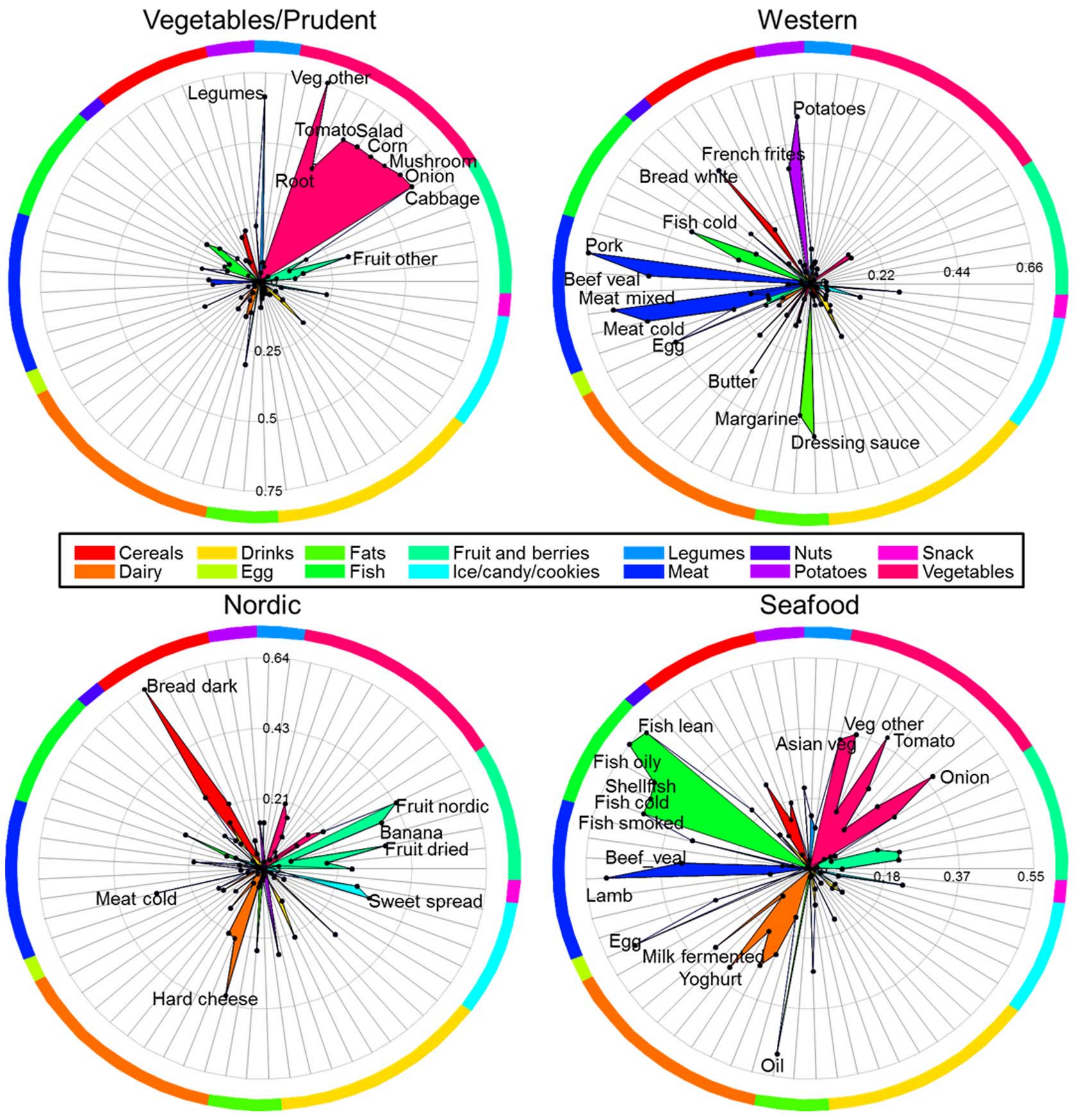

Figure 3. Spider plot(s) of pattern score vs. food item correlations. Upper left – *Vegetables/Prudent*, upper right – *Western*, lower left – *Nordic*, and lower right – *Seafood*. The colors refer to food item subgroups. Labels are only shown for food items with a correlation coefficient above 0.3.
doi:10.1371/journal.pone.0093644.g003

marine n-3 fatty acids [25,26], which in three recent trials have been consistently found to be able to prolong gestation or reduce early or very early preterm birth [27–29]. A RCT in Oslo found that if Norwegian women shifted to a Mediterranean type diet that promoted fish intake, the preterm rate fell [19]. This is consistent with the associations suggested by our Seafood diet. However, two ensuing observational studies, based on the large Danish and Norwegian national birth cohorts, respectively, attempted to replicate the findings of the Oslo trial by constructing exposure groups that mimicked the diet regimens used in the trial, possibly due to imprecision in defining the exposure [17,18]. While the findings of the Oslo trial could not be consistently replicated, the Norwegian cohort found a reduce risk of preterm birth with maternal fish intake.

Our study was not without limitations. Dietary data was collected using a validated FFQ, but may nonetheless be subject to measurement error. However, given the prospective nature of our analyses we expect any measurement errors to have been non-differential, attenuating the strength of our results towards the null. We only capture patterns which contributed significantly to the overall degree of variation. Hence, minor patterns only account for a small part of the population based on a small subset of variables e.g. ethnical or regional eating patterns, and would not have been captured by PCA. Furthermore, although we adjusted for many relevant confounders we cannot exclude residual confounding by excluded or mismeasured confounders. It is however reassuring that the association between Western diet and induced preterm birth was unaffected after adjustment for confounders as a Western-type diet is generally associated with attributes of an unhealthy lifestyle, including smoking.

Our study has notable strengths, including a prospective design and a large number of observations. We were able to use detailed dietary data to identify dietary patterns in our population using standard and accepted methodology. While dietary patterns are subject to the underlying consumption patterns in a population and may not be transferable across populations, they are nonetheless useful in describing intake in any given population. Furthermore, people tend to eat according to patterns and

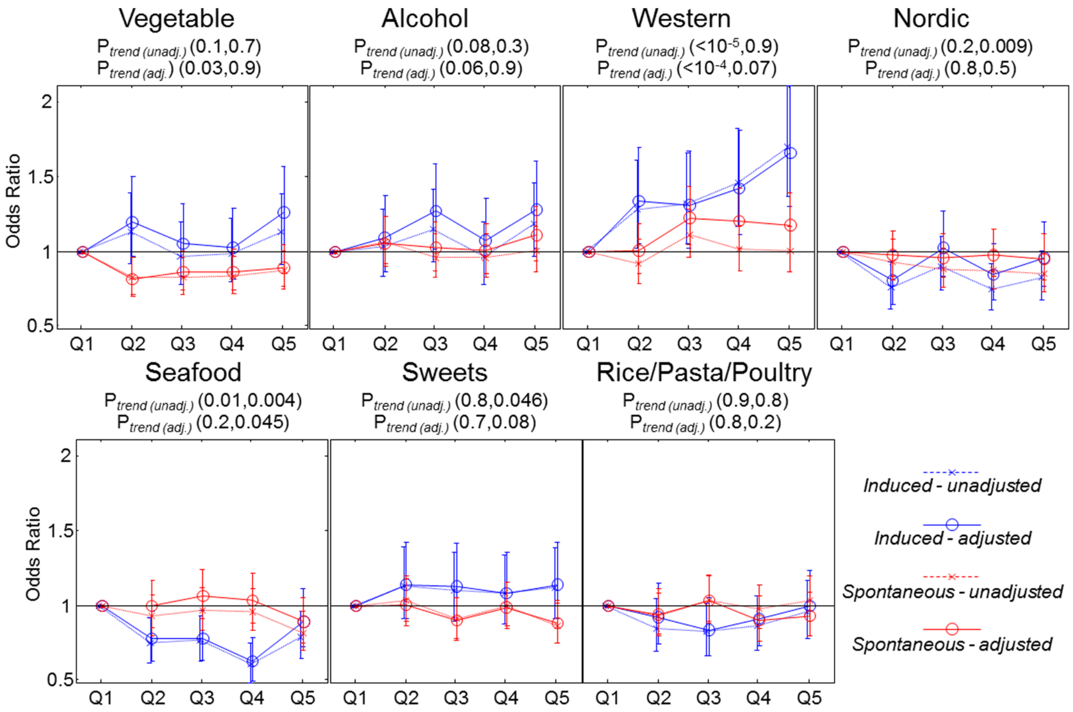

Figure 4. Odds ratio and 95% CI (unadjusted and adjusted for relevant confounders: maternal age, maternal height, pre-pregnancy BMI, parity, civil status, socioeconomic status and smoking during pregnancy) for preterm birth (spontaneous and induced) for each dietary pattern. Reference level is the lowest quintile. *P*-values in parenthesis are for induced- and spontaneous preterm birth respectively.
doi:10.1371/journal.pone.0093644.g004

Table 1. The associations between maternal covariates and preterm birth.

	**Spontaneous #cases = 1756, #controls = 57267**		**Induced #cases = 895, #controls = 57267**	
	**OR (CI 0.95)**	**P-value**	**OR (CI 0.95)**	**P-value**
Civil status (coupled/married compared to single)	0.98 (0.69–1.39)	0.9	0.97 (0.60–1.57)	0.9
Height (per 10 cm)	0.76 (0.70–0.83)	$<10^{-9}$	0.89 (0.79–0.99)	0.04
Nonsmoker	1	0.19	1	0.002
Occasionally smoker	0.87 (0.75–1.02)		0.97 (0.79–1.21)	
≥15 cigarettes/day	1.01 (0.86–1.18)		1.42 (1.17–1.73)	
High level proficiencies	1	0.015	1	0.32
Medium level proficiencies	1.12 (0.91–1.37)		1.03 (0.78–1.36)	
Skilled	1.13 (0.91–1.40)		1.01 (0.75–1.35)	
Students	1.12 (0.89–1.41)		0.90 (0.65–1.25)	
Unskilled	1.25 (1.01–1.54)		1.06 (0.79–1.42)	
Unemployed	1.47 (1.16–1.87)		1.28 (0.93–1.76)	
Age≤20	1	0.13	1	<0.001
21≤Age≤39	1.45 (0.86–2.44)		2.55 (0.94–6.89)	
Age≥40	2.12 (1.03–4.39)		6.24 (2.05–19.05)	
BMI≤18.5	1	<0.001	1	0.09
18.6≤BMI≤24.9	0.75 (0.60–0.93)		0.88 (0.63–1.23)	
25.0≤BMI≤29.9	0.65 (0.51–0.83)		1.04 (0.73–1.48)	
30.0≤BMI≤34.9	0.93 (0.70–1.23)		1.11 (0.73–1.68)	
BMI≥35.0	0.96 (0.66–1.39)		1.37 (0.83–2.28)	
Nulliparous	1	$<10^{-10}$	1	$<10^{-7}$
Primiparous	0.48 (0.42–0.54)		0.63 (0.54–0.75)	
Multiparous	0.40 (0.34–0.49)		0.70 (0.56–0.87)	

The marginal effect on preterm birth is calculated by a multivariate model with confounders/covariates and quintilized pattern scores for Western-type diet as predictor. Results are from stratification of the cases into spontaneous and induced births respectively.
doi:10.1371/journal.pone.0093644.t001

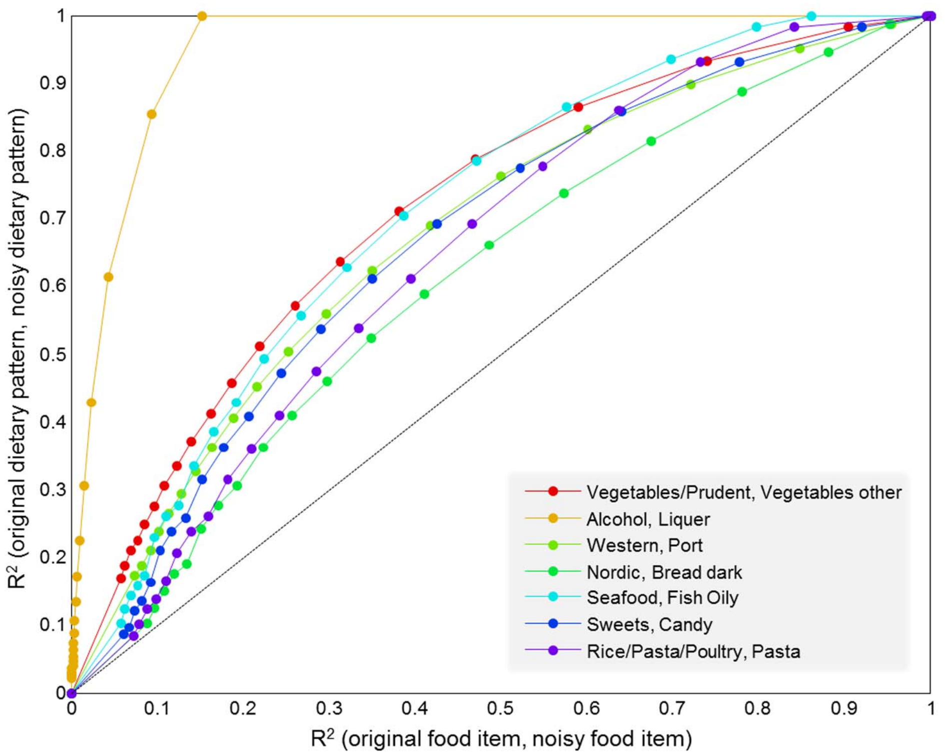

Figure 5. Comparison of the effect of random error in raw data and derived dietary factors. For each level of error (Signal to noise ratio = (∞, …, 0.1)) the correlation (R^2) between matched pairs of components from original model and model build on data plus Gaussian noise (y-axis) are matched with the correlation between the original data and the original data plus error for the most influential variable of the respective component (x-axis). The results are the average over 100 repetitions.
doi:10.1371/journal.pone.0093644.g005

repetitive clusters of foods and these may therefore be useful not only in relation to disease risk but also in making dietary recommendations. The dietary information used in this study derives from participant-completed food questionnaires. Although these are completed with support of detailed guidance, such data are subjected to a high degree of uncertainty and possibly bias. The foundation of dietary patterns via PCA relies on the covariance structure in the data, i.e. the correlation between different food items, and is hence less sensitive to unstructured registration error than the individual food items. However, biased registrations e.g. by overreporting, inflate the dietary factors and possibly bias association results based on these measures. In order to emphasize the effect of unstructured noise in the data, a small example is presented in **Figure 5**. Briefly, a seven component model based on the study data was compared with a seven component model based on the study data plus Gaussian error. **Figure 5** shows that the dietary patterns are more robust to the effect of unstructured noise compared to the individual food items.

In this study on dietary patterns we used novel visual tools to describe and characterize complex dietary data. We furthermore related these patterns to preterm birth and found a direct association between a Western-type diet and induced preterm birth, a finding which should be investigated further in future studies. We emphasize the use and utility of the visual tools applied to these analyses for future studies on dietary patterns. We also believe that these tools can be applied beyond dietary factors to other environmental and occupational exposures as well as clinical and physiological markers clustered around certain 'disease profiles'. We therefore suggest and encourage their use to facilitate communication of complex results to the research community and public.

Supporting Information

Appendix S1 Dietary pattern analysis, factor modelling and principal component analysis – some details.
(DOCX)

Figure S1 Cartoon of splitting dietary information into a common part reflected by dietary patterns (here *Western*, *Vegetables* and *Seafood*) and a person unique part which cannot be ascribed to general phenomena.
(TIF)

Figure S2 Spider plot(s) of pattern score vs. food item correlations. Upper left – *Alcohol*, upper right – *Sweets*, lower– *Rice/Pasta/Poultry*. The colors refer to food item subgroups. Labels are only shown for food items with a correlation coefficient above 0.3.
(TIF)

File S1 Tables of food composition of the seven dietary patterns, reflected as average intake in g/day (mean and standard deviation) within pattern score quintiles.
(PDF)

Author Contributions

Conceived and designed the experiments: MAR EM TIH SFO. Performed the experiments: MAR. Analyzed the data: MAR. Contributed reagents/materials/analysis tools: MAR. Wrote the paper: MAR EM TIH. Designed the software used in the analysis: MAR.

References

1. Hu FB (2002). Dietary pattern analysis: a new direction in nutritional epidemiology. Current opinion in lipidology 13: 3–9.
2. Northstone K, Emmett PM (2008) A comparison of methods to assess changes in dietary patterns from pregnancy to 4 years post-partum obtained using principal components analysis. The British journal of nutrition 99: 1099–1106.
3. Cuco G, Fernandez-Ballart J, Sala J, Viladrich C, Iranzo R, et al. (2006) Dietary patterns and associated lifestyles in preconception, pregnancy and postpartum. European journal of clinical nutrition 60: 364–371.
4. Torjusen H, Lieblein G, Naes T, Haugen M, Meltzer HM, et al. (2012) Food patterns and dietary quality associated with organic food consumption during pregnancy; data from a large cohort of pregnant women in Norway. BMC public health 12:612.
5. Okubo H, Miyake Y, Sasaki S, Tanaka K, Murakami K, et al. (2011) Nutritional adequacy of three dietary patterns defined by cluster analysis in 997 pregnant Japanese women: the Osaka Maternal and Child Health Study. Public health nutrition 14: 611–621.
6. Brantsaeter AL, Haugen M, Samuelsen SO, Torjusen H, Trogstad L, et al. (2009) A dietary pattern characterized by high intake of vegetables, fruits, and vegetable oils is associated with reduced risk of preeclampsia in nulliparous pregnant Norwegian women. The Journal of nutrition 139: 1162–1168.
7. Zhang C, Schulze MB, Solomon CG, Hu FB (2006) A prospective study of dietary patterns, meat intake and the risk of gestational diabetes mellitus. Diabetologia 49: 2604–13.
8. Wolff CB, Wolff HK (1995) Maternal eating patterns and birth weight of Mexican American infants. Nutrition and health 10: 121–134.
9. Okubo H, Miyake Y, Sasaki S, Tanaka K, Murakami K, et al. (2012) Maternal dietary patterns in pregnancy and fetal growth in Japan: the Osaka Maternal and Child Health Study. The British journal of nutrition 107: 1526–1533.
10. Thompson JM, Wall C, Becroft DM, Robinson E, Wild CJ, et al. (2010) Maternal dietary patterns in pregnancy and the association with small-for-gestational-age infants. The British journal of nutrition 103: 1665–1673.
11. Vujkovic M, Steegers EA, Looman CW, Ocke MC, van der Spek PJ, et al. (2009) The maternal Mediterranean dietary pattern is associated with a reduced risk of spina bifida in the offspring. BJOG : an international journal of obstetrics and gynaecology 116: 408–415.
12. Lange NE, Rifas-Shiman SL, Camargo CA Jr, Gold DR, Gillman MW, et al. (2010) Maternal dietary pattern during pregnancy is not associated with recurrent wheeze in children. The Journal of allergy and clinical immunology 126: 250–255.
13. Chatzi L, Apostolaki G, Bibakis I, Skypala I, Bibaki-Liakou V, et al. (2007) Protective effect of fruits, vegetables and the Mediterranean diet on asthma and allergies among children in Crete. Thorax 62: 677–683.
14. Miyake Y, Okubo H, Sasaki S, Tanaka K, Hirota Y (2011) Maternal dietary patterns during pregnancy and risk of wheeze and eczema in Japanese infants aged 16–24 months: the Osaka Maternal and Child Health Study. Pediatric allergy and immunology : official publication of the European Society of Pediatric Allergy and Immunology 22: 734–741.
15. Cole ZA, Gale CR, Javaid MK, Robinson SM, Law C, et al. (2009) Maternal dietary patterns during pregnancy and childhood bone mass: a longitudinal study. Journal of bone and mineral research : the official journal of the American Society for Bone and Mineral Research 24: 663–668.
16. Musselman JR, Jurek AM, Johnson KJ, Linabery AM, Robison LL, et al. (2011) Maternal dietary patterns during early pregnancy and the odds of childhood germ cell tumors: A Children's Oncology Group study. American journal of epidemiology 173: 282–291.
17. Haugen M, Meltzer HM, Brantsaeter AL, Mikkelsen T, Østerdal ML, et al. (2008) Mediterranean-type diet and risk of preterm birth among women in the Norwegian Mother and Child Cohort Study (MoBa): a prospective cohort study. Acta obstetricia et gynecologica Scandinavica 87: 319–324.
18. Mikkelsen TB, Osterdal ML, Knudsen VK, Haugen M, Meltzer HM, et al. (2008) Association between a Mediterranean-type diet and risk of preterm birth among Danish women: a prospective cohort study. Acta obstetricia et gynecologica Scandinavica 87: 325–330.
19. Khoury J, Henriksen T, Christophersen B, Tonstad S (2005) Effect of a cholesterol-lowering diet on maternal, cord, and neonatal lipids, and pregnancy outcome: a randomized clinical trial. American journal of obstetrics and gynecology 193: 1292–1301.
20. Olsen SF, Mikkelsen TB, Knudsen VK, Orozova-Bekkevold I, Halldórsson TI, et al. (2007) Data collected on maternal dietary exposures in the Danish National Birth Cohort. Paediatr Perinat Epidemiol 21: 76–86.
21. Willett WC (1998) Nutritional Epidemiology, 2nd ed. New York, USA: Oxford University Press. pp. 273–301.
22. Kaiser HF (1958) The varimax criterion for analytic rotation in factor analysis. Psychometrika 23: 187–200.
23. Berkowitz GS, Blackmore-Prince C, Lapinski RH, Savitz DA (1998) Risk factors for preterm birth subtypes. Epidemiology (Cambridge, Mass) 9: 279–285.
24. Roberts CL, Ford JB, Algert CS, Antonsen S, Chalmers J, et al. (2011) Population-based trends in pregnancy hypertension and pre-eclampsia: an international comparative study. BMJ open 1(1):e000101.
25. Olsen SF, Hansen HS, Sorensen TI, Secher N, Jensen B, et al. (1986) Intake of marine fat, rich in (n-3)-polyunsaturated fatty acids, may increase birthweight by prolonging gestation. Lancet 328(8503): 367–369.
26. Olsen SF, Sorensen JD, Secher NJ, Hedegaard M, Henriksen TB, et al. (1992) Randomised controlled trial of effect of fish-oil supplementation on pregnancy duration. Lancet 339(8800): 1003–1007.
27. Carlson SE, Colombo J, Gajewski BJ, Gustafson KM, Mundy D, et al. (2013) DHA supplementation and pregnancy outcomes. The American journal of clinical nutrition 97: 808–815.
28. Hauner H, Much D, Vollhardt C, Brunner S, Schmid D, et al. (2012) Effect of reducing the n-6:n-3 long-chain PUFA ratio during pregnancy and lactation on infant adipose tissue growth within the first year of life: an open-label randomized controlled trial. The American journal of clinical nutrition 95: 383–394.
29. Makrides M, Gibson RA, McPhee AJ, Collins CT, Davis PG., et al. (2009) Neurodevelopmental outcomes of preterm infants fed high-dose docosahexaenoic acid: a randomized controlled trial. JAMA: the journal of the American Medical Association 301: 175–182.
